# Integrated analysis reveals critical glycolytic regulators in hepatocellular carcinoma

**DOI:** 10.1186/s12964-020-00539-4

**Published:** 2020-06-23

**Authors:** Chenying Lu, Shiji Fang, Qiaoyou Weng, Xiuling Lv, Miaomiao Meng, Jinyu Zhu, Liyun Zheng, Yumin Hu, Yang Gao, Xulu Wu, Jianting Mao, Bufu Tang, Zhongwei Zhao, Li Huang, Jiansong Ji

**Affiliations:** 1 Key Laboratory of Imaging Diagnosis and Minimally Invasive Intervention Research, the Fifth Affiliated Hospital of Wenzhou Medical University /Affiliated Lishui Hospital of Zhejiang University/The Central Hospital of Zhejiang Lishui, Lishui, 323000 PR China; 2 Department of Radiology, the Fifth Affiliated Hospital of Wenzhou Medical University /Affiliated Lishui Hospital of Zhejiang University/The Central Hospital of Zhejiang Lishui, Lishui, 323000 PR China; 3 School of Materials Science and Engineering, Shanghai Key Laboratory of D&A for Metal-Functional Materials, Tongji University, Shanghai, 201804 PR China

**Keywords:** Liver cancer, Tumor metabolism, Energy metabolism, SPP1

## Abstract

**Background:**

Cancer cells primarily utilize aerobic glycolysis for energy production, a phenomenon known as the Warburg effect. Increased aerobic glycolysis supports cancer cell survival and rapid proliferation and predicts a poor prognosis in cancer patients.

**Methods:**

Molecular profiles from The Cancer Genome Atlas (TCGA) cohort were used to analyze the prognostic value of glycolysis gene signature in human cancers. Gain- and loss-of-function studies were performed to key drivers implicated in hepatocellular carcinoma (HCC) glycolysis. The molecular mechanisms underlying Osteopontin (OPN)-mediated glycolysis were investigated by real-time qPCR, western blotting, immunohistochemistry, luciferase reporter assay, and xenograft and diethyl-nitrosamine (DEN)-induced HCC mouse models.

**Results:**

Increased glycolysis predicts adverse clinical outcome in many types of human cancers, especially HCC. Then, we identified a handful of differentially expressed genes related to HCC glycolysis. Gain- and loss-of-function studies showed that OPN promotes, while SPP2, LECT2, SLC10A1, CYP3A4, HSD17B13, and IYD inhibit HCC cell glycolysis as revealed by glucose utilization, lactate production, and extracellular acidification ratio. These glycolysis-related genes exhibited significant tumor-promoting or tumor suppressive effect on HCC cells and these effects were glycolysis-dependent. Mechanistically, OPN enhanced HCC glycolysis by activating the αvβ3-NF-κB signaling. Genetic or pharmacological blockade of OPN-αvβ3 axis suppressed HCC glycolysis in xenograft tumor model and hepatocarcinogenesis induced by DEN.

**Conclusions:**

Our findings reveal crucial determinants for controlling the Warburg metabolism in HCC cells and provide a new insight into the oncogenic roles of OPN in HCC.

**Video Abstract**

## Background

Hepatocellular carcinoma (HCC), the main type of liver cancer, is an increasingly prevalent clinical problem. HCC is the fifth most common cancer worldwide and the third most frequent cause of cancer-related death worldwide [1]. Many risk factors contributes for HCC initiation, such as infection with either hepatitis B virus or hepatitis C virus, nonalcoholic steatohepatitis, alcoholic cirrhosis, and exposure to environmental toxins [2, 3]. Despite many progresses in the diagnosis and treatment of HCC, the prognosis remains dismal due to high rates of recurrence and intrahepatic metastasis after surgical resection [4]. Therefore, uncovering the mechanisms underlying HCC progression to improve clinical outcomes and to develop better therapeutic strategies is of paramount importance [5].

Accumulating evidences have shown that reprogrammed energy metabolism is a hallmark of tumor cells and a critical contributor to tumor development [6, 7]. Different from most normal cells, cancer cells metabolize glucose to lactate even in the presence of sufficient oxygen, a phenomenon termed aerobic glycolysis, also known as “Warburg effect” [8]. In the past decade, there is a considerable resurgence of interest in the oncogenic role of aerobic glycolysis in cancers. The aerobic glycolysis is characterized by a much higher rate of glucose uptake, consumption and lactate release in cancer cells. Increased glycolysis facilitates cancer cells to rapid utilization of glucose to produce abundant ATP. Through the pentose phosphate pathway, glucose metabolism provides cancer cells with abundant cellular buildings for biosynthetic pathways, including nucleotides, lipids, and nonessential amino acids. Therefore, the glycolysis benefits both bioenergetics and biosynthesis necessary for cancer cell survival and rapid proliferation [8, 9]. Moreover, glycolysis-derived lactate can lead to an acidic tumor microenvironment, which is profoundly implicated in tumor progression by modulating tumor metastasis and immune response [10]. Thus, targeting tumor glycolysis holds distinct promise for development of therapeutic strategies for cancer patients [11]. However, the molecular mechanism for the Warburg effect in HCC is far from explored.

Increased glycolysis is commonly viewed as a consequence of oncogenic event that drives the aggressive phenotypes of cancer cells. During liver malignant transformation, a series of genetic alterations will be selected, including activation of oncogenes and inactivation of tumor suppressors. For example, highly expressed PARP14 can regulate PKM2 activity to promote the Warburg effect and cell survival in HCC [12]; phosphorylation of ATG4B can impair mitochondrial activity and enhance the Warburg effect [13]; inactivation of HELLS leads to metabolic reprogramming and reverses the Warburg effect [14]. Notably, loss of FBP1 facilitates aggressive features of HCC cells through the Warburg effect [15]. Several glycolytic enzymes are also upregulated in cirrhotic livers and significantly associated with an increased risk for developing HCC [16]. Previously, Jiang et al. have documented the glycolysis gene expression profiling in HCC and identified six mRNAs (DPYSL4, HOMER1, ABCB6, CENPA, CDK1, and STMN1) significantly associated with overall survival for HCC [17]. Although several modulators involved in HCC glycolysis have been revealed, a comprehensive characterization of glycolysis-related genes is timely needed.

In this study, by leveraging large-scale molecular profiles from The Cancer Genome Atlas (TCGA) cohort, we identify many differentially expressed glycolysis-related genes in HCC. Functional experiments show that Osteopontin (OPN) is a positive regulator, while SPP2, LECT2, SLC10A1, CYP3A4, HSD17B13, and IYD are negative regulator for HCC glycolysis. OPN, encoded by *SPP1* (secreted phosphoprotein1) gene, plays a crucial role in HCC initiation and progression and can be acts as a biomarker for HCC [18–22]. Previously, intense investigations have well characterized the oncogenic roles of OPN in HCC. Here, we provide new evidence that OPN is also profoundly implicated in HCC glycolysis by activating the αvβ3-NF-κB signaling.

## Materials and methods

### Data mining

The RNA-sequencing data of HCC and corresponding adjacent non-tumor liver tissues were downloaded from The Cancer Genome Atlas (TCGA, https://gdc.cancer.gov/) database. The glycolysis score was calculated based on the mRNA expression value of *SLC2A1*, *HK2*, *GPI*, *PFKL*, *ALDOA*, *GAPDH*, *PGK1*, *PGAM1*, *ENO1*, *PKM2*, and *LDHA*. Differentially expressed genes were identified by estimating an exact test *P*-value.

### Cell culture and reagents

The human HCC cell lines (Huh7, HCC-LM3, SMCC-7721, MHCC-97H, Hep3B, and MHCC-97H), NIH3T3 cells, and MEFs were obtained from American Type Culture Collection (ATCC, UK). All cells were cultured with Dulbecco’s modified Eagle’s medium (DMEM, Gibco, Shanghai) supplemented with 10% fetal bovine serum (FBS), 2 mM L-glutamine, 1% penicillin-streptomycin (Sigma, USA), and maintained in a humidified incubator with 5% CO_2_. All cells were tested negative for cross-contamination of other human cells and mycoplasma contamination. The glycolysis inhibitor 2-Deoxy-D-glucose (2-DG) was obtained from Sigma Aldrich (D6134, St Louis, MO, USA). Galactose was purchased from Sigma-Aldrich (G5388, St Louis, MO, USA). The recombinant OPN protein was purchased from R&D systems (1433-OP-050, Shanghai, China).

### Cell transfection

The genetic modulation in this study was performed by lentiviral system. The shRNA for OPN and scramble control was obtained from Genepharma Biotechnology (Shanghai, China). The full length sequences of genes for overexpression strategy were synthesized from Sangon Biotech (Shanghai, China). For transfection, cells were grown on 6-well culture plates with 70–80% confluence. Transfection was performed with Lipofectamine 2000 (#11668019, Invitrogen, Carlsbad, CA, USA) according to the procedures of the manufacturer. Lentiviral particles were packaged in 293 T cells using second-generation packaging plasmids psPAX2 (Addgene plasmid 12,260) and pMD2.G (Addgene plasmid 12,259). The cells supernatant was collected 72 h after transfection. The virus was concentrated using ultracentrifuge at 20,000 rpm for 3 h and added to target cells with 5 μg/ml of polybrene (Millipore, USA).

### Detection of lactate and glucose level

HCC cells seeded into 6-well plates at a density of 5 × 10^5^ cells were cultured in fresh phenol red-free medium and incubated for 24 h before the culture medium were collected. The lactate and glucose levels in the culture medium were determined by the glucose assay kit (Abcam, Cambridge, MA, USA) and lactate assay kit according to the manufacturer’s instruction (Biovision), and normalized with cell number.

### Measurement of extracellular acidification rate

The Seahorse XF96 Flux Analyser (Seahorse Bioscience) was used to monitor extracellular acidification rate (ECAR) according to the manufacturer’s instructions. In brief, HCC cells seeded into 96-well plates at a density of 1.5 × 10^4^ per well were cultured in complete medium. Before experiment, the culture medium was replaced by test buffer (Seahorse Bioscience). For ECAR test, cells were incubated with buffered medium followed by a sequential injection of 10 mM glucose, 1 mM oligomycin and 50 mM 2-DG. The final glycolytic ability was normalized to cell number.

### Colony formation assay

For anchorage-dependent growth assay, cells were seeded onto a 6-well plate (1000 cells/well). The culture medium was replaced every 2 days. After 10–12 days, the colonies were fixed with 4% paraformaldehyde and stained with 0.5% crystal violet. The numbers of colonies greater than 100 μm in diameter were counted.

### Enzyme linked immunosorbent assay (ELISA) analysis

HCC cell supernatants were collected, cleared by centrifugation and used immediately. The amount of supernatants used was normalized to cell number. The OPN ELISA kits were purchased from R&D systems (DOST00, Shanghai, China) and used according to the manufacturer’s instructions.

### Quantitative real-time PCR

Total RNA was extracted from HCC cell lines using Trizol Reagent (Invitrogen, CA, USA). Total RNA (1 μg) was reverse transcribed using PrimeScript® reverse transcriptase Master Mix (TaKaRa, Dalian, China) according to the manufacturer’s instructions. Real-time qPCR was carried out on an Applied Biosystems 7500 apparatus using SYBR-Green Master mix (TaKaRa, Dalian, China). All samples including the template controls were tested in triplicate. The relative expression of target transcripts was normalized to human *ACTB* gene transcripts. The delta Ct method was used to calculate the relative expression. The primer sequences used in this study were provided in Supplementary Table 1.

### Western blotting analysis

Total protein was extracted from HCC cell lines using a total protein extraction buffer (Beyotime, Shanghai, China) with phosphatase and proteinase inhibitors (1 mM EDTA, 1 mM sodium orthovanadate, 10 mM sodium pyrophosphate, 100 mM NaF, 10 mg/ml leupeptin, 10 units/ml aprotinin, 1 mM phenylmethylsulfonyl fluoride) and protein concentration was measured using a BCA Protein Assay Kit (Pierce Biotechnology, USA). Protein samples were separated by sodium dodecyl sulfate-polyacrylamide gel electrophoresis (SDS-PAGE) and transferred onto polyvinylidene fluoride membranes. After blocked with 5% non-fat milk/TBST, the membrane was incubated with the primary antibody against OPN (Abcam, ab8448), Akt (Cell Signaling Technology, #4685), p-Akt (Cell Signaling Technology, #4060), Erk1/2 (Cell Signaling Technology, #4695), p-Erk1/2 (Cell Signaling Technology, #9101), p65 (Cell Signaling Technology, #8242), p-p65 (Cell Signaling Technology, #3033), and β-actin (Abcam, ab8227). The signals were acquired after incubation with the species-specific secondary antibodies using an Odyssey Infrared Imaging System (LI-COR, Lincoln, NE).

### Immunohistochemistry

Paraffin-embedded liver tissues were mounted on silicon-coated slides, dewaxed, and rehydrated, and antigen retrieval was then performed. The slides were incubated with anti-OPN antibody (1:100; Abcam; ab8448)). Quantitation of immunoreactivity was calculated based on the intensity and number of positive cells.

### Luciferase reporter assay

HCC cells were transfected with NF-κB-driven luciferase plasmid together with the pRL-TK, which contains the herpes simplex virus thymidine kinase (HSK-TK) promoter to provide low to moderate levels of Renilla luciferase expression by using Lipofectamine 2000 (#11668019, Invitrogen, Carlsbad, CA, USA). After 24 h, the cells were collected and subjected to luciferase activity detection using the Dual Luciferase Reporter Assay System (Promega, USA).

### Animal study

Six-week-old male BALB/c nude mice (Experimental Animal Centre, SIBS) were used in our study. Wild type or modified HCC-LM3 cells were injected subcutaneously into the right flank of these mice to establish xenograft model. Mouse experiments were conducted in accordance with the NIH Guidelines for the Care and Use of Laboratory Animals. The study procedures were approved by the Fifth Affiliated Hospital of Wenzhou Medical University. For pharmacological inhibition experiment, mice were randomly divided into two groups when the tumor volume reached to 200 mm^3^. Mice were treated with Cilengitide (5 mg/kg) three times a week, while control group was treated with saline with 0.01% DMSO. Tumor volume (mm^3^) was estimated by the formula: volume = length × width^2^/2. The tumor volumes data are presented as means ± SD. For DEN-induced HCC model, male C57BL/6-Spp1^tm1Blh^ (OPN^−/−^) (OPN KO) mice were purchased from Jackson Laboratory (Bar Harbor, ME, USA). The mice at 2 weeks old were injected with 25 mg/kg of DEN (Sigma, Shanghai, China) or vehicle intraperitoneally once to induce hepatic carcinogenesis. Mice in the control groups were not subjected to DEN injection. All mice were sacrificed at 35 weeks after the injection of DEN.

### Statistical analysis

All experiments were conducted independently for three times. Statistical analyses were performed using the SPSS 16.0 (SPSS, Chicago, IL, USA) or Prism 5.0 (GraphPad, La Jolla, CA, USA). The differences between groups were analyzed using Student’s t-test or one-way ANOVA with Dunnett’s multiple comparisons. The Spearman’s rank correlation coefficient and chi-square tests were used to analysis the correlation between indicated parameters. Kaplan-Meier survival analysis was performed to compare patient survival data. *P* < 0.05 was considered to be statistically significant. *P < 0.05, **P < 0.01, and ***P < 0.001.

## Results

### Prognostic analyses of glycolysis gene signature in human cancers

**Fig. 1:**
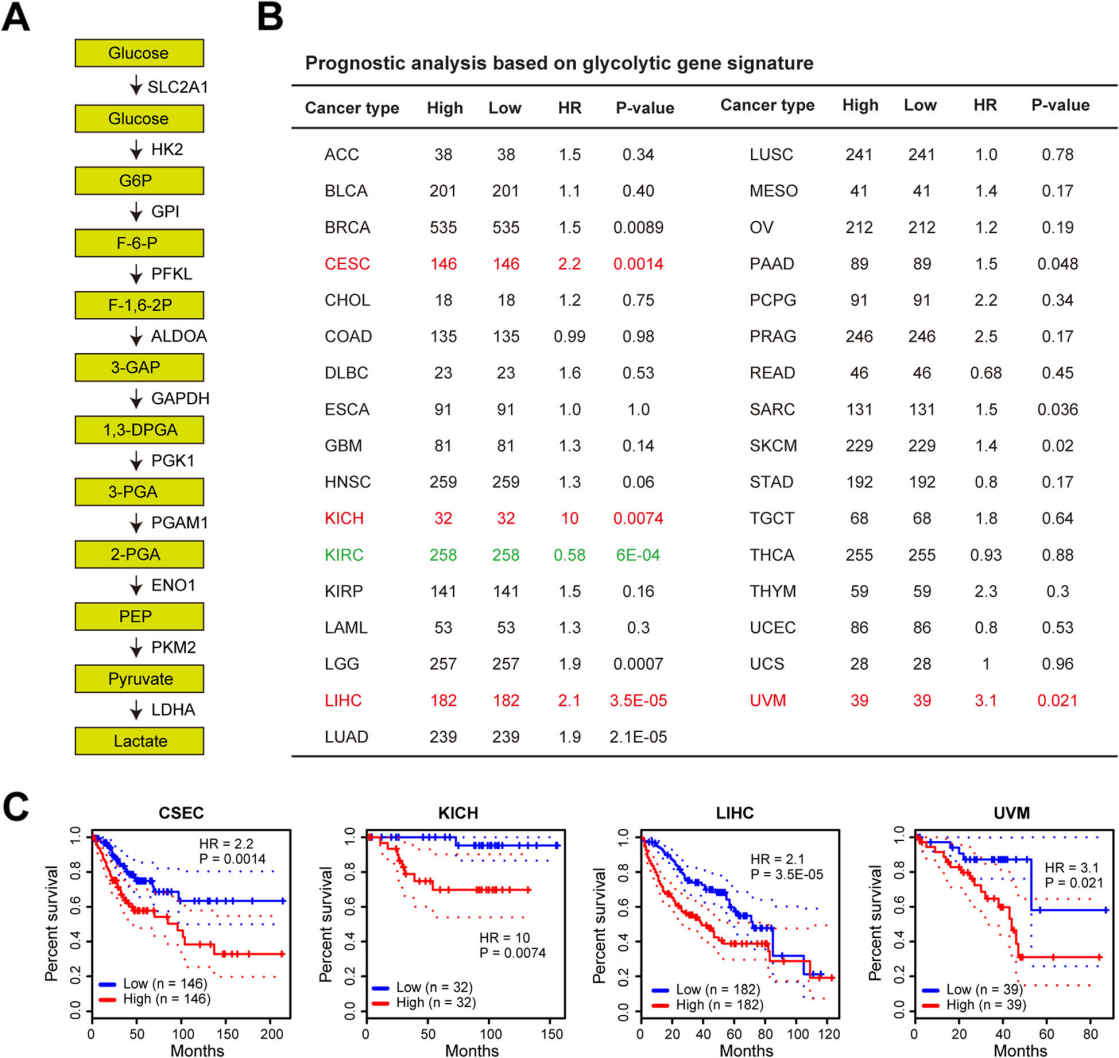
Prognostic analyses of glycolysis signature in human cancers. **a** Summary of the genes in glycolysis pathway. **b** The glycolysis score of TCGA tumor samples was calculated based on the expression level of glycolytic genes. Based on the median glycolysis score, the prognostic value of glycolysis gene signature in each tumor type was analyzed. HR: hazard ratio. **c** Kaplan-Meier curves of glycolysis gene signature in CSEC, KICH, LIHC, and UVM. Abbreviations for TCGA cancer types are available at http://gepia2.cancer-pku.cn/#dataset

Emerging evidences suggest that increased glycolysis contributes to tumor malignant phenotypes and patients’ poor prognosis [9, 23]. More than ten genes encoding the glucose transporter and glycolytic enzymes are directly responsible for the aerobic glycolysis [24] (Fig. 1a). To identify the correlation between glycolysis and patients’ clinical outcome in human cancers, we calculated a glycolysis score of each tumor sample from The Cancer Genome Atlas (TCGA) cohort based on the expression level of glycolytic components (glucose transporter and glycolytic enzymes). Using the median glycolysis as a cutoff, we analyzed the prognostic value of glycolysis gene signature across 33 types of human cancers (Fig. 1b). As a result, tumor glycolysis was closely correlated with poor prognosis in many types of human cancers, especially for cervical squamous cell carcinoma and endocervical adenocarcinoma (CSES), kidney chromophobe (KICH), liver hepatocellular carcinoma (LIHC), and uveal melanoma (UVM), which had a hazard ratio lager than 2 (Fig. 1c). Specifically, increased glycolysis predicted a better clinical outcome in KIRC patients (Fig. 1b).

### Identification of differentially expressed genes for HCC glycolysis

**Fig. 2:**
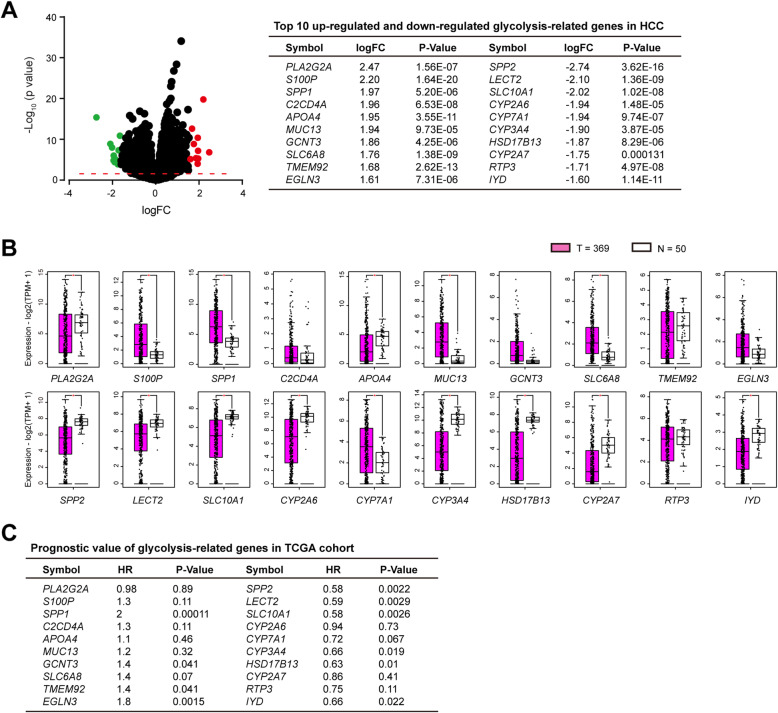
Identification of differentially expressed glycolysis-related genes in HCC. **a** Volcano plot of differentially expressed genes (DEGs) in HCC. Red dot represents the top 10 up-regulated genes, while green dot represents the top 10 updown-regulated genes. Detailed information of DEGs was shown in the right panel. FC: fold change. **b** The expression pattern of DEGs in HCC tumor tissues (*n* = 369) and normal liver tissues (*n* = 50). **c** Prognostic analysis of DEGs in TCGA HCC cohort. The median expression was used as a cutoff. *P < 0.05

**Fig. 3:**
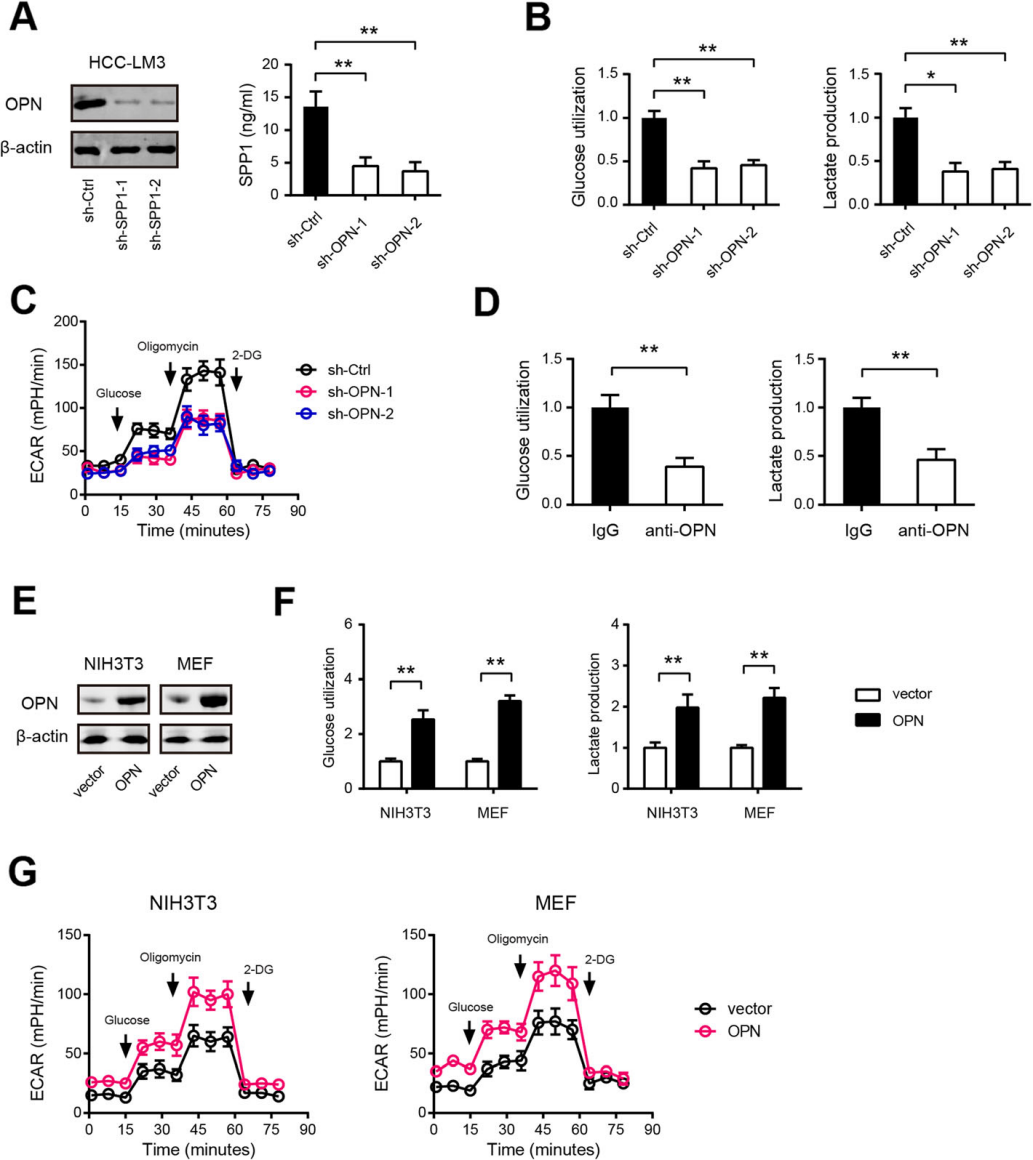
OPN promotes the Warburg effect in HCC cells. **a** The knockdown efficiency of OPN in HCC-LM3 cells was measured by Western blotting and ELISA. **b** Effects of OPN knockdown on the glucose uptake and lactate production in HCC-LM3 cells (*n* = 3). **c** The extracellular acidification rate (ECAR) in sh-OPN and sh-Ctrl HCC-LM3 cells was measured by Seahorse analyzer (*n* = 5). **d** Effects of OPN blockade on the glucose uptake and lactate production in HCC-LM3 cells (*n* = 3). **e** The overexpression efficiency of OPN in NIH3T3 cells and MEFs was measured by Western blotting. **f** Effects of OPN overexpression on the glucose uptake and lactate production in NIH3T3 cells and MEFs (*n* = 3). **g** Effects of OPN overexpression on ECAR in NIH3T3 cells and MEFs were measured by Seahorse analyzer (*n* = 5). *P < 0.05 and **P < 0.01

Aerobic glycolysis in cancer cells are largely mediated by activated oncogenes and/or loss of tumor suppressors. In this study, we aimed to uncover the critical modulators responsible for HCC glycolysis. To achieve this, we compared the gene expression pattern among TCGA HCC samples with high and low glycolysis score and identified a handful of differentially expressed genes (DEGs). The top 10 up-regulated and down-regulated DEGs were shown in Fig. 2a. In the top 10 up-regulated genes, *S100P* and *SPP1* were highly expressed in HCC tissues compared with normal liver tissues, whereas *PLA2G2A* and *APOA4* expression were down-regulated in HCC (Fig. 2b); highly expressed *SPP1*, *TMEM92*, and *EGLN3* predicted a poor prognosis in HCC patients (Fig. 2c and Supplementary Fig. 1). In the top 10 down-regulated genes, *SPP2*, *LECT2*, *SLC10A1*, *CYP2A6*, *CYP3A4*, *HSD17B13*, *CYP2A7*, and *IYD* expression were significantly down-regulated, while *CYP7A1* expression was up-regulated in tumor tissues in comparison to normal liver tissues (Fig. 2b); notably, *SPP2*, *LECT2*, *SLC10A1*, *CYP3A4*, *HSD17B13*, and *IYD* predicted a better prognosis in HCC patients (Fig. 2c and Supplementary Fig. 1). Combined these data above, we hypothesized that SPP1, SPP2, LECT2, SLC10A1, CYP3A4, HSD17B13, and IYD might be critical modulators for HCC glycolysis. Indeed, there genes had a close correlation with the glycolysis gene signature (Supplementary Fig. 2). Consistent with the results in HCC-LM3, NIH3T3 cells and MEFs expressing OPN exhibited increased glycolysis (Fig. 3e-g), excluding the possibility that the observed differences in glycolysis could be attributed to a cell type-specific phenomenon.

### Certification of glycolysis-related genes in HCC cells

**Fig. 4:**
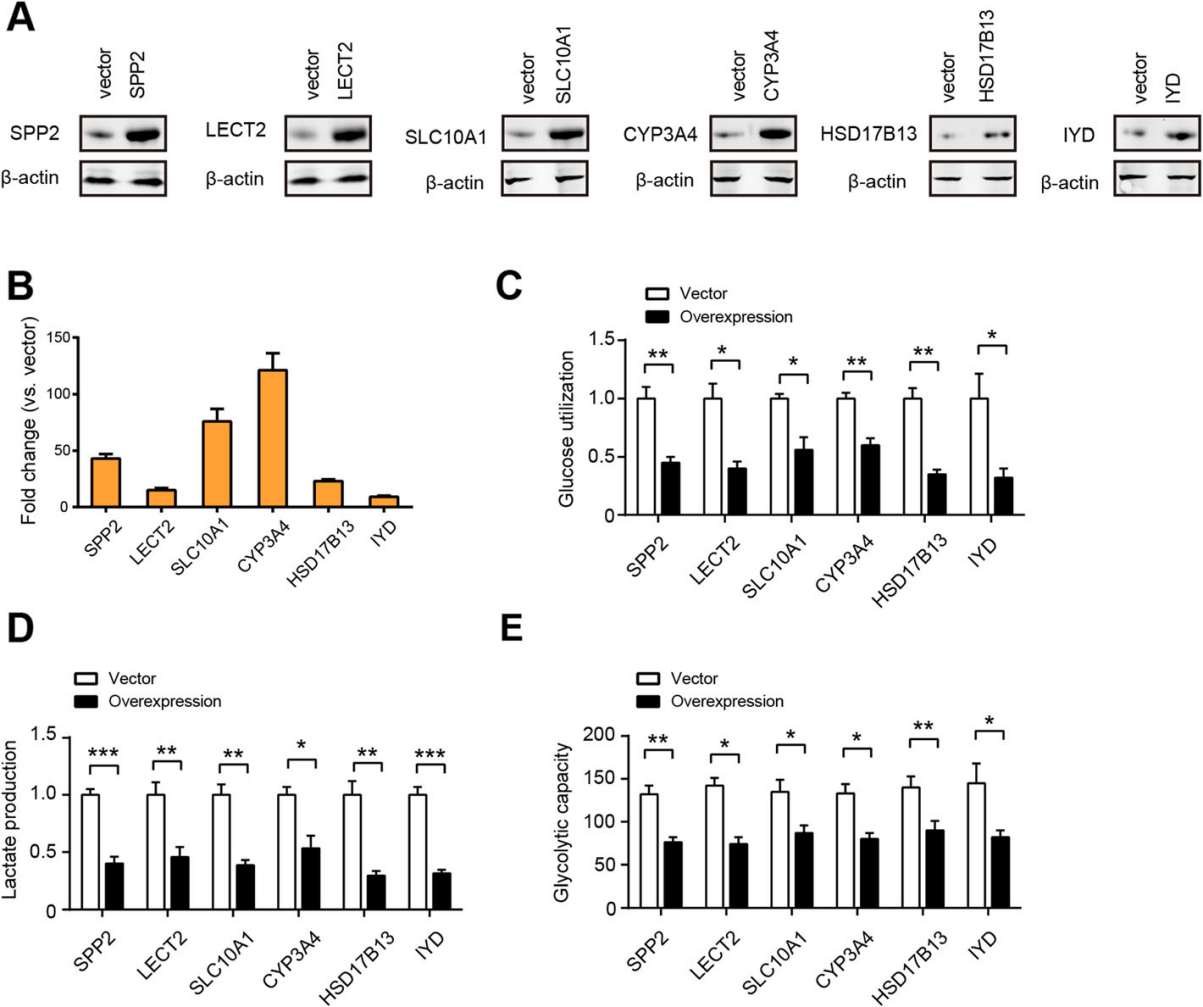
Certification of the negative regulators of HCC glycolysis. **a** Western blotting showed the overexpression efficiency of SPP2, LECT2, SLC10A1, CYP3A4, HSD17B13, and IYD in Huh7 cells. **b** Real-time qPCR analysis showed the overexpression efficiency of SPP2, LECT2, SLC10A1, CYP3A4, HSD17B13, and IYD in Huh7 cells (*n* = 3). **c-e** Measurement of SPP2, LECT2, SLC10A1, CYP3A4, HSD17B13, or IYD overexpression on the glucose utilization (**f**, *n* = 3), lactate production (**g**, *n* = 3) and ECAR (**h**, *n* = 5) in Huh7 cells. *P < 0.05, **P < 0.01, and ***P < 0.001

Previous studies have well documented diverse aggressive phenotypes induced by OPN (SPP1) in HCC, including tumor growth, metastasis, epithelial-mesenchymal transition, stemness, drug resistance, immunosuppression, and etc. [25–28]. However, no report is available about its regulatory role in HCC glycolysis. To test whether up-regulated OPN contributes to HCC glycolysis, loss-of-function study was performed in HCC-LM3 cells, which preserve higher OPN at both mRNA and secreted level (Supplementary Fig. 3A and 3B). Two specific shRNA against OPN led to significant reduction in OPN protein level (Fig. 3a). In HCC-LM3 cells, genetic silencing of OPN significantly inhibited glucose utilization and lactate production (Fig. 3b). In addition, treatment with recombinant OPN protein increased glucose utilization and lactate production in Huh7 and Hep3B cells in a dose-dependent manner (Supplementary Fig. 3C and D). To further confirm this observation, Seahorse Extracellular Flux analysis was performed to determine the cellular bioenergetic activity. As a result, the extracellular acidification ratio (ECAR) was markedly reduced by OPN knockdown (Fig. 3c). Moreover, blocking of OPN with its neutralizing antibody also attenuated the glycolytic ability of HCC-LM3 cells as revealed by reduced glucose uptake and lactate production (Fig. 3d). Consistent with the results in HCC-LM3, NIH3T3 cells and MEFs expressing OPN exhibited increased glycolysis (Fig. 3e-g), excluding the possibility that the observed differences in glycolysis could be attributed to a cell type-specific phenomenon. Similarly, we evaluated the potential regulatory role of SPP2, LECT2, SLC10A1, CYP3A4, HSD17B13, and IYD in HCC glycolysis by gain-of-function strategy. The overexpression efficiency of indicated genes was verified by western blotting (Fig. 4a) and real-time qPCR (Fig. 4b), respectively. As displayed in Fig. 4c-e, overexpression of SPP2, LECT2, SLC10A1, CYP3A4, HSD17B13, and IYD differentially suppressed HCC glycolytic capacity as demonstrated by reduced glucose uptake, lactate release, and ECAR.

### Effects of glycolysis-related genes on HCC tumor growth

**Fig. 5:**
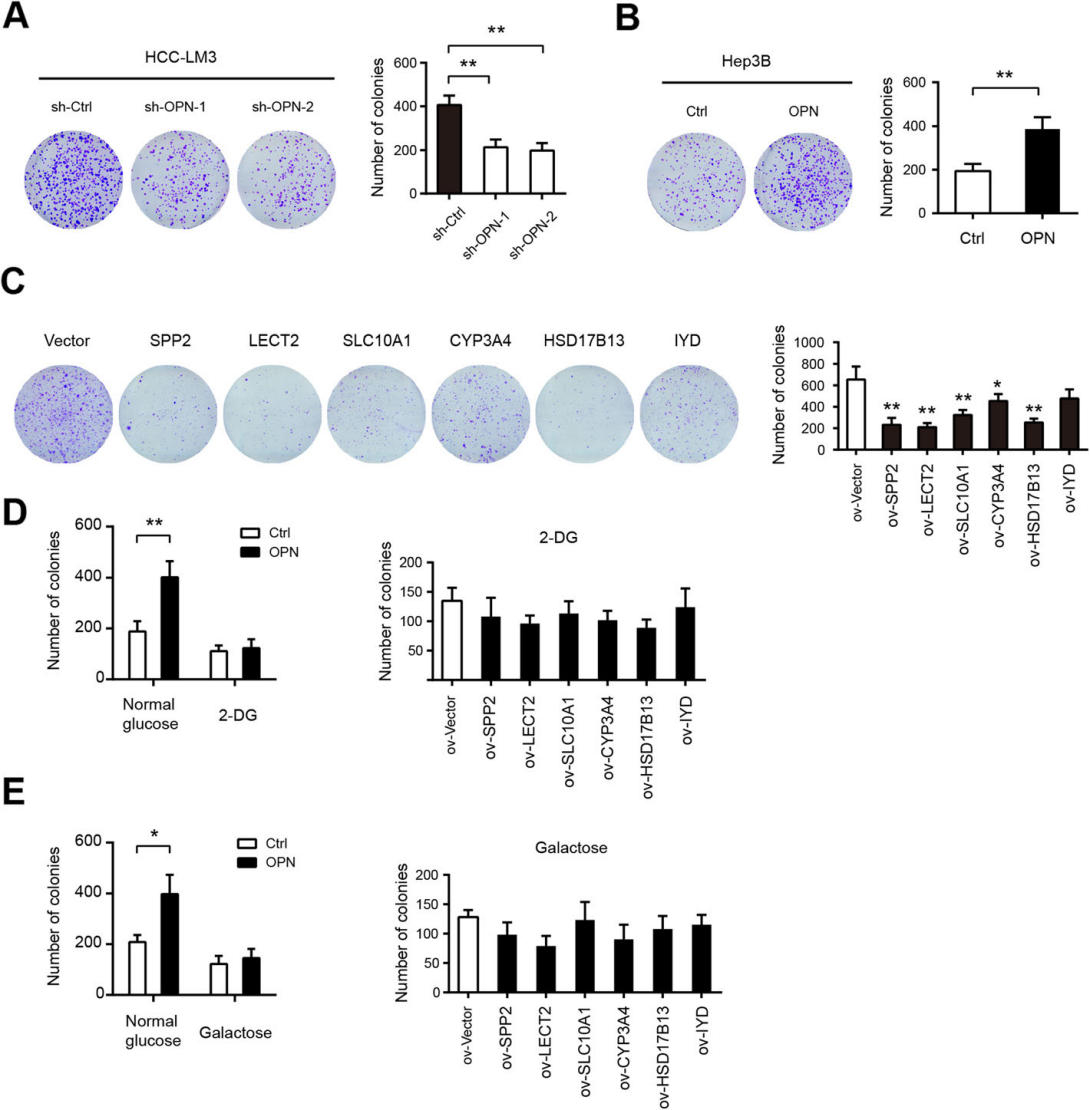
Effects of glycolysis-related genes on HCC tumor growth. **a** Colony formation assay showed that OPN knockdown or blockade inhibits HCC-LM3 cell proliferation (*n* = 3). **b** Colony formation assay for Huh3B cells treated with recombinant OPN protein (*n* = 3). **c** Colony formation assay showed that SPP2, LECT2, SLC10A1, CYP3A4, HSD17B13, or IYD overexpression inhibits Huh7 cell proliferation (*n* = 3). **d** The effects of glycolysis-related genes on HCC tumor growth in the presence or absence of 5 mM 2-DG (*n* = 3). **e** In the culture medium containing 25 mM glucose or galactose, the effects of glycolysis-related genes on HCC tumor growth were analyzed by clonogenic assay. *P < 0.05 and **P < 0.01

Warburg effect can provide abundant cellular blocks for cancer cell rapid proliferation [8]. Therefore, we investigated whether stimulating or inhibiting the Warburg effect is an important mechanism regulating HCC tumorigenesis by genetic modulation of OPN, SPP2, LECT2, SLC10A1, CYP3A4, HSD17B13, and IYD. The colony formation assay showed that OPN knockdown led to drastic reduction in HCC-LM3 cell anchorage-dependent growth (Fig. 5a), whereas recombinant OPN protein clearly facilitated Hep3B cell anchorage-dependent proliferation (Fig. 5b). Expectedly, overexpression of SPP2, LECT2, SLC10A1, CYP3A4, HSD17B13, and IYD diversely suppressed Huh7 cell anchorage-dependent growth (Fig. 5c). To further confirm this observation, we added 5 mM 2-Deoxy-D-glucose (2-DG) in the culture medium to inhibit glycolysis. The results showed that 2-DG greatly inhibited anchorage-dependent growth of HCC-LM3 and Huh7 cells (Fig. 5d). Notably, 2-DG largely compromised the effects induced by OPN recombinant protein or LECT2, SLC10A1, CYP3A4, HSD17B13, and IYD knockdown (Fig. 5d). Moreover, we replaced the glucose in the culture medium with galactose, which occurs at a much lower rate than glucose entry into glycolysis. Likewise, galactose mirrored the effect induced by 2-DG (Fig. 5e). Collectively, these data above strongly suggest that OPN, SPP2, LECT2, SLC10A1, CYP3A4, HSD17B13, and IYD in regulating the aerobic glycolysis affect HCC tumorigenesis.

### OPN promotes HCC glycolysis by modulating αvβ3-NF-κB signaling

**Fig. 6:**
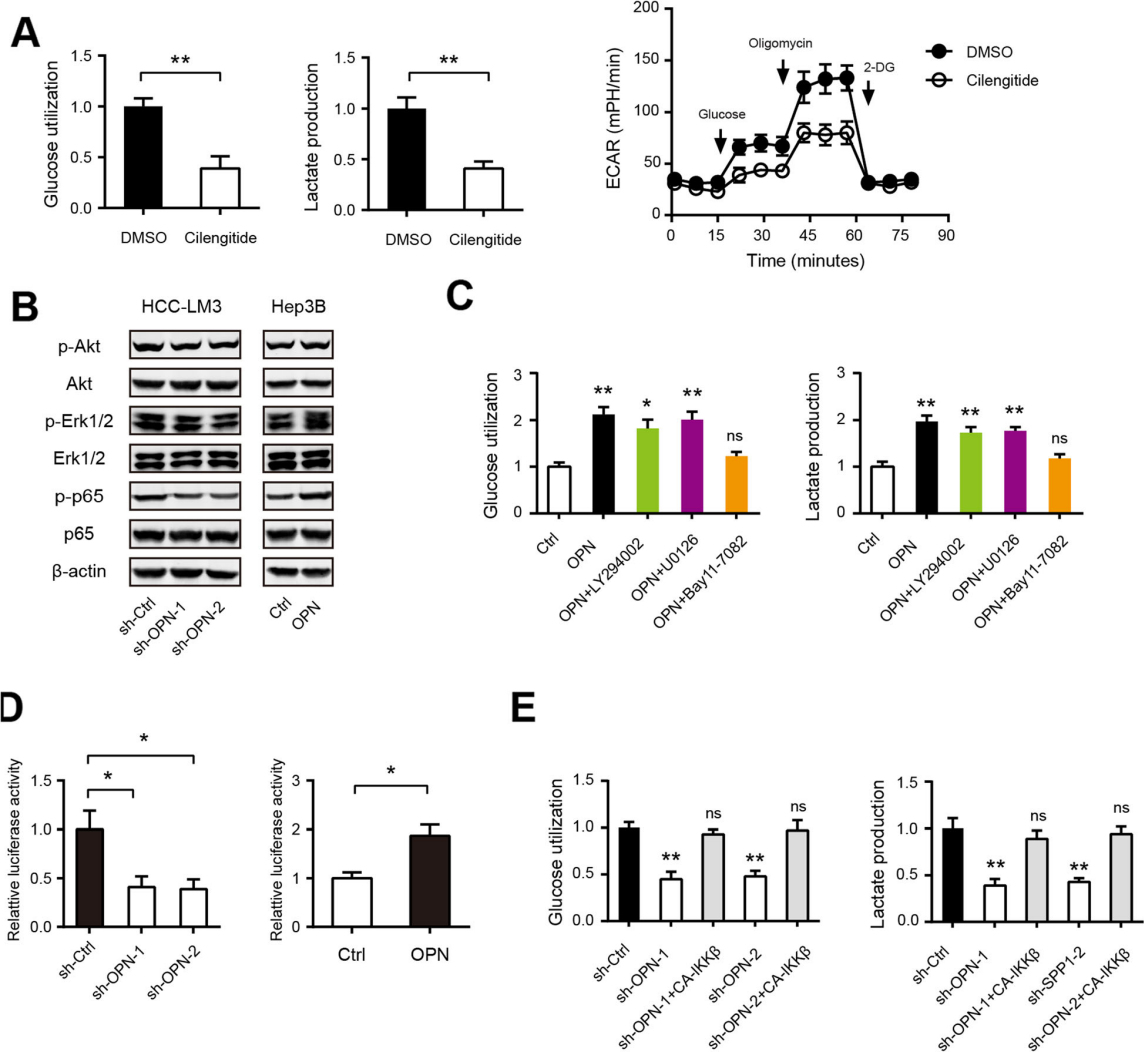
OPN promotes HCC glycolysis by modulating αvβ3-NF-κB signaling. **a** Blockade of integrin αvβ3 with Cilengitide inhibits glucose utilization (*n* = 3), lactate production (*n* = 3) and ECAR (*n* = 5) in HCC-LM3 cells. **b** Western blotting analysis the signaling pathway influenced by OPN. **c** Glucose utilization and lactate production in Hep3B cells upon treatment with OPN recombinant protein and indicated pathway inhibitors (*n* = 3). **d** Effect of OPN on the NF-κB activity in HCC cells (*n* = 3). **e** Effect of CA-IKKβ on the glucose uptake and lactate production in OPN-silenced HCC-LM3 cells (*n* = 3). *P < 0.05 and **P < 0.01; ns: not significant

Next, we pursued the mechanism by which OPN promotes HCC glycolysis. Integrin receptor αvβ3 is well documented as a critical receptor for OPN-mediated oncogenic activities in HCC [29]. Indeed, pharmacological inhibition of αvβ3 with Cilengitide significantly inhibited HCC-LM3 cell glucose utilization, lactate production, and ECAR (Fig. 6a). Upon binding to integrins and CD44 receptor family, OPN activates various signaling cascade, including PI3K/Akt, and MAPK, and nuclear factor kappa B (NF-κB) in HCC. Western blotting showed that OPN knockdown drastically inhibited the phosphorylation levels of p65, while the phosphorylation levels of Akt and Erk1/2 were slightly reduced. Consistently, OPN treatment significantly increased phosphorylation levels of p65 in Hep3B cells, indicating the prominent regulatory role of OPN in NF-κB activity (Fig. 6b). Interestingly, small molecule inhibitors against NF-κB (Bay11–7082) but not PI3K/Akt (LY294002) and MAPK (U0126) largely abrogated the glycolysis induced by OPN (Fig. 6c). By luciferase reporter assay, we found that OPN knockdown inhibited, whereas recombinant OPN protein increased NF-κB-responsive reporter activity in HCC cells (Fig. 6d). Notably, ectopic expression of constitutively active I-kappa B kinase complex β (CA-IKKβ), at least, to some extent, rescued the reduced glycolysis induced by OPN knockdown (Fig. 6e). Taken together, these data provide mechanism support for the regulatory role of OPN in HCC glycolysis by activating NF-κB signaling.

### Inhibition of OPN-αvβ3 axis suppresses HCC tumor growth and glycolyis

**Fig. 7:**
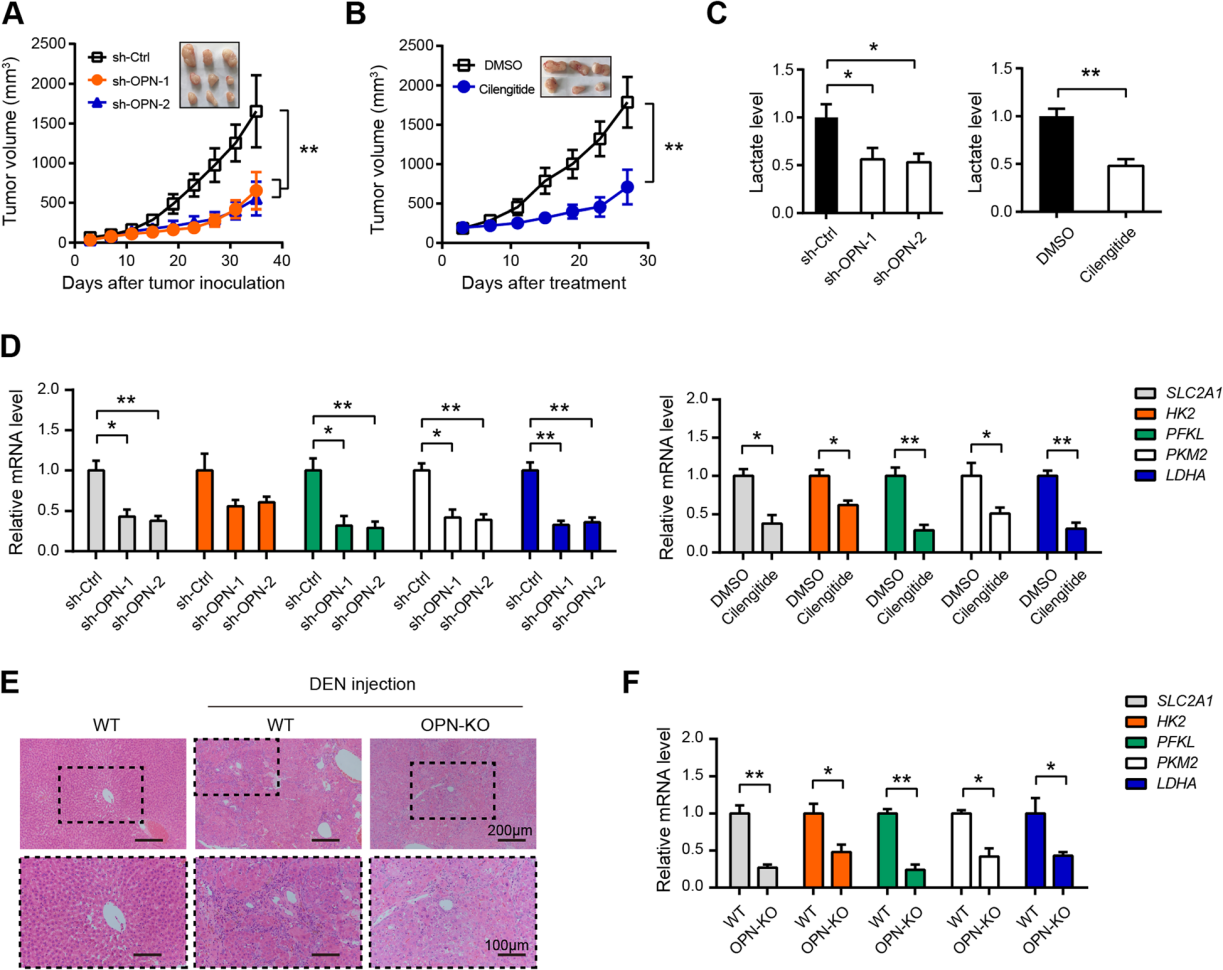
Inhibition of OPN-αvβ3 axis suppresses HCC tumor growth and glycolyis. **a** Tumor volume in sh-Ctrl and sh-OPN HCC-LM3 xenografts as indicated time point was measured (*n* = 5). **b** Effect of Cilengitide treatment on the tumor growth of HCC-LM3 xenografts (*n* = 5). **c** The lactate level in the tumor tissues from (**a**) and (**b**) was detected (*n* = 5). **d** The expression of glycolytic genes in the tumor tissues from (**a**) and (**b**) was analyzed by real-time qPCR (*n* = 5). **e** Hematoxylin and eosin staining in liver tissue samples from tumor-bearing WT and OPN-KO mice. **f** The expression of glycolytic genes in liver tissue samples from tumor-bearing WT and OPN-KO mice was analyzed by real-time qPCR (*n* = 5). *P < 0.05 and **P < 0.01

Furthermore, we evaluated the therapeutic potential of targeting OPN-αvβ3 axis in HCC. Consistent with previous report, tumor growth of xenografts formed from sh-OPN HCC-LM3 cells was much slower compared with that formed from sh-Ctrl HCC-LM3 cells (Fig. 7a). Moreover, pharmacological blockade of αvβ3 with Cilengitide remarkably inhibited tumor growth of HCC-LM3 xenografts (Fig. 7b). By measuring the lactate level in xenograft tumor tissues, we found that both OPN knockdown and αvβ3 inhibition reduced lactate concentration in vivo (Fig. 7c). Moreover, real-time qPCR analysis showed that OPN knockdown significantly down-regulated the expression of glucose transporter (SLC2A1) and glycolytic enzymes (HK2, PFKL, PKM2, and LDHA) (Fig. 7d). Similar result was also noticed in the pharmacological inhibition studies (Fig. 7d). Moreover, we generated DEN-induced HCC model in OPN-KO and wide type (WT) mice. As shown in Fig. 7e, the nodules from the OPN KO mice at 35 weeks were composed of well-differentiated tumor cells or vacuolated cells that formed trabeculae or nests, whereas the nodules from the WT mice showed a sessile and solid growth pattern. Real-time qPCR resulted revealed that expression of the glycolytic enzymes was markedly reduced in liver tissues of OPN-KO mice compared to that in WT mice (Fig. 7f). Collectively, these data indicate that targeting OPN-αvβ3 axis is sufficient to block HCC tumor growth and glycolysis.

### Expression pattern of OPN in clinical samples

**Fig. 8:**
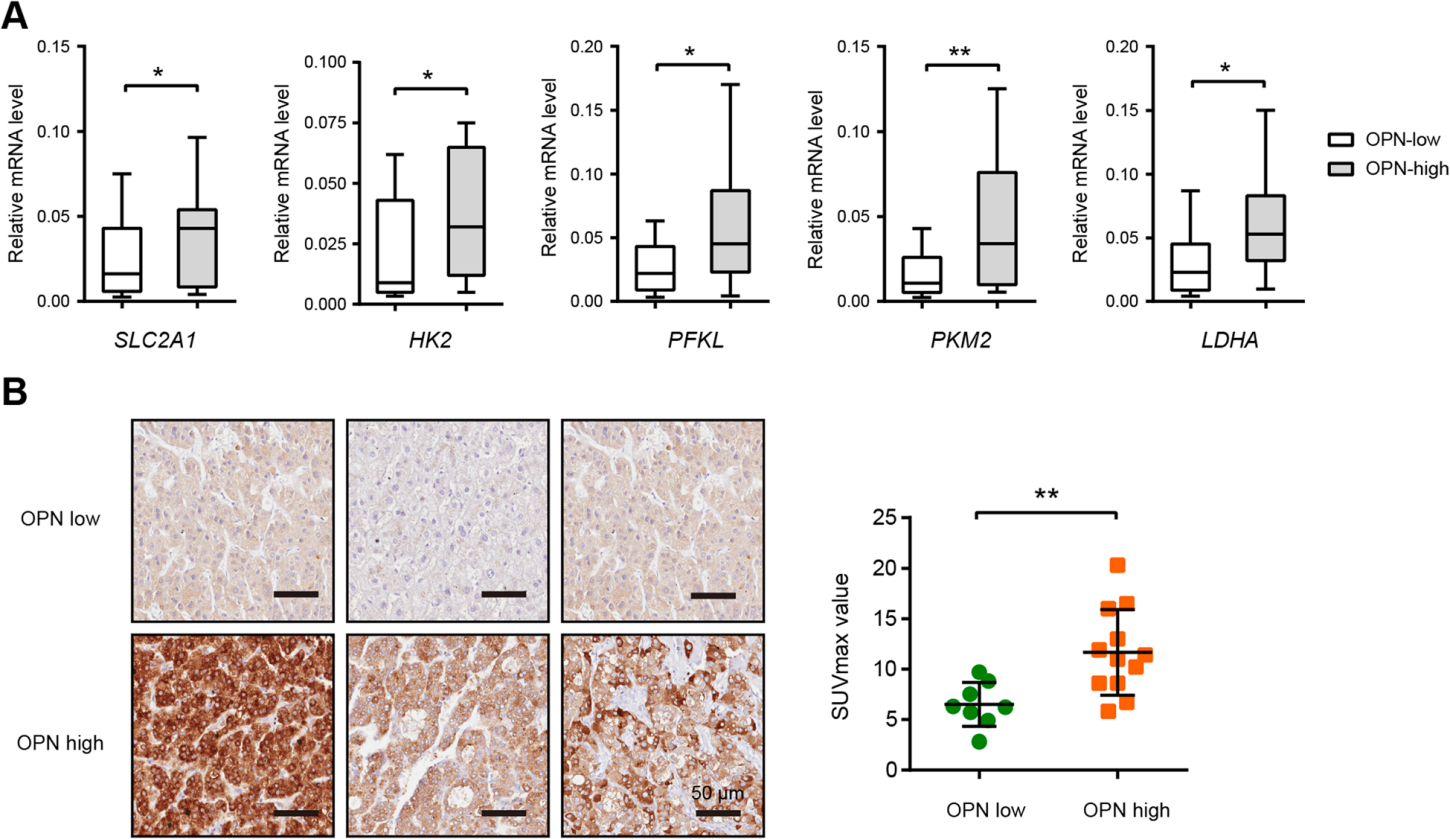
Expression pattern of OPN in clinical samples. **a** The expression of glycolytic genes in human HCC tissue samples with high OPN (*n* = 10) and low OPN (*n* = 20) expression was analyzed by real-time qPCR. **b** Representative photographs of OPN expression in HCC tumor tissues; scale bar: 50 μm. The correlation between OPN expression and the SUVmax value was analyzed. *P < 0.05 and **P < 0.01

Consistent with previous reports, OPN is highly expressed in human HCC samples and correlated a poor overall survival rate and disease-free survival rate in HCC patients (Supplementary Fig. 4). In this study, we further investigated the correlation between OPN and the Warburg metabolism in clinical HCC samples. In a cohort of 60 HCC patients, the glycolytic enzymes were highly expressed in OPN-high samples compared to OPN-low HCC samples (Fig. 8a). Moreover, in a cohort of 20 HCC patients who received preoperative ^18^F-FDG PET/CT, we found that the SUVmax was much higher in the group with high expression of OPN (Fig. 8b). This result further supports the critical regulatory of the OPN in HCC glycolysis.

## Discussion

Many molecular targeted therapies and diagnostic methods have been developed for the treatment of HCC; however, the clinical outcome of HCC is still unsatisfactory. Recent data show that increased glycolysis is closely associated with poor prognosis in cancer patients, and targeting cancer glycolysis metabolism represents an attractive strategy for cancer therapy. Therefore, it is very important to uncover critical modulators that regulate HCC glycolysis. In this study, we found that tumor samples with high glycolysis gene signature had a significant reduced overall survival time in many cancer types, especially in HCC. By leveraged TCGA HCC RNA-seq data, we identified seven differentially expressed genes (OPN, SPP2, LECT2, SLC10A1, CYP3A4, HSD17B13, and IYD) associated with HCC glycolysis. In addition, these genes had a close correlation with the glycolysis score and patients’ prognosis in HCC. Notably, none of these genes have been reported to play a role in the regulation in cancer metabolism. Our experimental data demonstrated glycolytic alterations induced by these genes are linked to tumor growth. The functional validation of glycolysis-related genes provides candidate therapeutic targets and associated biomarkers for this deadly disease.

OPN is a multifunctional secreted phosphoprotein with intense studies in liver disease, including acute liver injury, autoimmune and viral hepatitis, alcoholic liver diseases, non-alcoholic fatty fiver diseases, liver fibrosis and HCC [25]. Importantly, OPN is involved in numerous oncogenic activities in HCC, including the cell survival, proliferation, stemness, angiogenesis, invasion, and metastasis [21, 28, 30, 31]. High OPN level is significantly associated with poor overall survival and OPN expression is positively associated with stage and tumor size [32]. OPN is also markedly increased in the plasma of HCC patients and emerged as a diagnostic biomarker that improves AFP performance in HCC surveillance among patients with HBV or HCV-related cirrhosis [33, 34]. In line with previous report, we found that OPN is overexpressed in HCC tissues and predicts a poor prognosis. By loss-of-function study, we certified that OPN is an important glycolysis regulator in HCC. Consistently, blockade of OPN with neutralizing antibody significantly suppressed HCC cell glycolytic activity. Interesting, tumor glycolysis-induced acidic microenvironment can activate hepatic stellate cells, which produce OPN under acidic condition and in turn promote the migration of HCC cells [35]. Previously, Shi et al. showed that OPN-a isoform increases the cellular glucose levels, and OPN-c utilizes this glucose to generate energy in breast cancer cells [36]. These data together suggest the regulatory role of OPN in tumor glycolysis. In HCC, OPN acts through multiple adhesion receptor binding motifs including integrins and CD44 receptors to promote tumor progression and metastasis [29, 37]. In this study, we revealed that αvβ3-NF-κB signaling is responsible for OPN-induced glcolysis in HCC. Given other integrins and CD44 receptor are also profoundly implicated in reprogrammed energy metabolism of cancer cells, we cannot fully rule out their contributions in HCC [38, 39]. OPN has been well studied as a potential therapeutic target in HCC treatment [40]. Consistent observation with in HCC cells with lower OPN level, glycolytic capacity was also increased by overexpression of OPN in normal MEF and NIH3T3 cells, suggesting the regulatory role of OPN on glycolysis is not cell type-specific phenomenon. Given that OPN is highly expressed in the HCC tumor tissues and can be merely detected in normal liver, there should be a therapeutic window for targeting OPN for HCC treatment. Therefore, our study further broadens the mechanism regarding the therapeutic potential by targeting cancer glucose metabolism.

Apart from OPN, the role of LECT2, SLC10A1, CYP3A4, and HSD17B13 are also characterized in HCC. LECT2 (Leukocyte cell-derived chemotoxin 2) is originally identified as a chemotactic factor for neutrophils and stimulates the growth of chondrocytes and osteoblasts and acts as a tumor suppressor in HCC by multiple mechanisms, such as inactivation of MET, controlling inflammatory monocytes, and inhibition of VEGF165/VEGFR2-dependent signaling [41–43]. Down-regulated SLC10A1 is correlated with poor post-surgery survival rate and larger tumor tissue mass in HCC patients and ectopic expression of NTCP significantly suppresses HCC cell proliferation [44]. Down-regulation of CYP3A4 gene is an independent predictor for HCC early recurrence [45]. HSD17B13 expression in peritumoral tissues is associated with worse recurrence free survival and overall survival of HCC patients and the HSD17B13 rs72613567 loss of function variant is protective of HCC development in patients with alcoholic liver disease [46, 47]. The expression pattern and functional role of IYD in cancers are not known. In this study, we for the first time showed that LECT2, SLC10A1, CYP3A4, and HSD17B13 can suppress HCC tumor growth by inhibiting Warburg effect. However, the detailed mechanism by which these genes regulate glycolysis warrants further investigations.

## Conclusion

Our comprehensive analysis provides essential insights into HCC glycolysis and uncovers several critical glycolysis-related genes. OPN promotes HCC glycolysis through activation of αvβ3-NF-κB signaling. LECT2, SLC10A1, CYP3A4, HSD17B13, and IYD can suppress HCC cell proliferation by down-regulating glycolytic flux. Therefore, our results open new avenues for designing potential therapeutic strategies for human HCC.

## Supplementary information

**Additional file 1 Supplementary Fig. 1** The prognostic value of glycolysis-related genes in HCC. **Supplementary Fig. 2** The correlation between glycolysis-related genes and the glycolysis signature. **Supplementary Fig. 3** Expression pattern and roles of OPN in HCC cells. **Supplementary Fig. 4** Expression pattern and prognostic value of OPN in HCC tissues. **Supplementary Table 1**: Primers used in this study**. Supplementary Table 2:** Group information.

## Data Availability

Source data and reagents are available from the corresponding author upon reasonable request.

## Abbreviations

TCGA: The Cancer Genome Atlas (TCGA)
HCC: Hepatocellular carcinoma
ATCC: American Type Culture Collection
DMEM: Dulbecco’s modified Eagle’s medium
FBS: Fetal bovine serum
OPN: Osteopontin
ECAR: Extracellular acidification rate
CSES: Endocervical adenocarcinoma
KICH: Kidney chromophobe
LIHC: Liver hepatocellular carcinoma
UVM: Uveal melanoma
DEGs: Differentially expressed genes
2-DG: 2-Deoxy-D-glucose
LECT2: Leukocyte cell-derived chemotoxin 2
