# Correction to: Integrated analysis reveals critical glycolytic regulators in hepatocellular carcinoma

**DOI:** 10.1186/s12964-020-00674-y

**Published:** 2020-10-22

**Authors:** Chenying Lu, Shiji Fang, Qiaoyou Weng, Xiuling Lv, Miaomiao Meng, Jinyu Zhu, Liyun Zheng, Yumin Hu, Yang Gao, Xulu Wu, Jianting Mao, Bufu Tang, Zhongwei Zhao, Li Huang, Jiansong Ji

**Affiliations:** 1 Key Laboratory of Imaging Diagnosis and Minimally Invasive Intervention Research, the Fifth Affiliated Hospital of Wenzhou Medical University/Affiliated Lishui Hospital of Zhejiang University/The Central Hospital of Zhejiang Lishui, Lishui, 323000 People’s Republic of China; 2 Department of Radiology, the Fifth Affiliated Hospital of Wenzhou Medical University/Affiliated Lishui Hospital of Zhejiang University/The Central Hospital of Zhejiang Lishui, Lishui, 323000 People’s Republic of China; 3 School of Materials Science and Engineering, Shanghai Key Laboratory of D&A for Metal-Functional Materials, Tongji University, Shanghai, 201804 People’s Republic of China

## Correction to: Cell Commun Signal (2020) 18:97 https://doi.org/10.1186/s12964-020-00539-4

Following publication of the original article [1], it was noticed that two duplicate images in Figs. 3e and 8b and were reported. The correct images are presented in this correction article and the correction does not change the conclusion of this paper. The authors would like to apologize for any inconvenience caused.

**Fig. 3:**
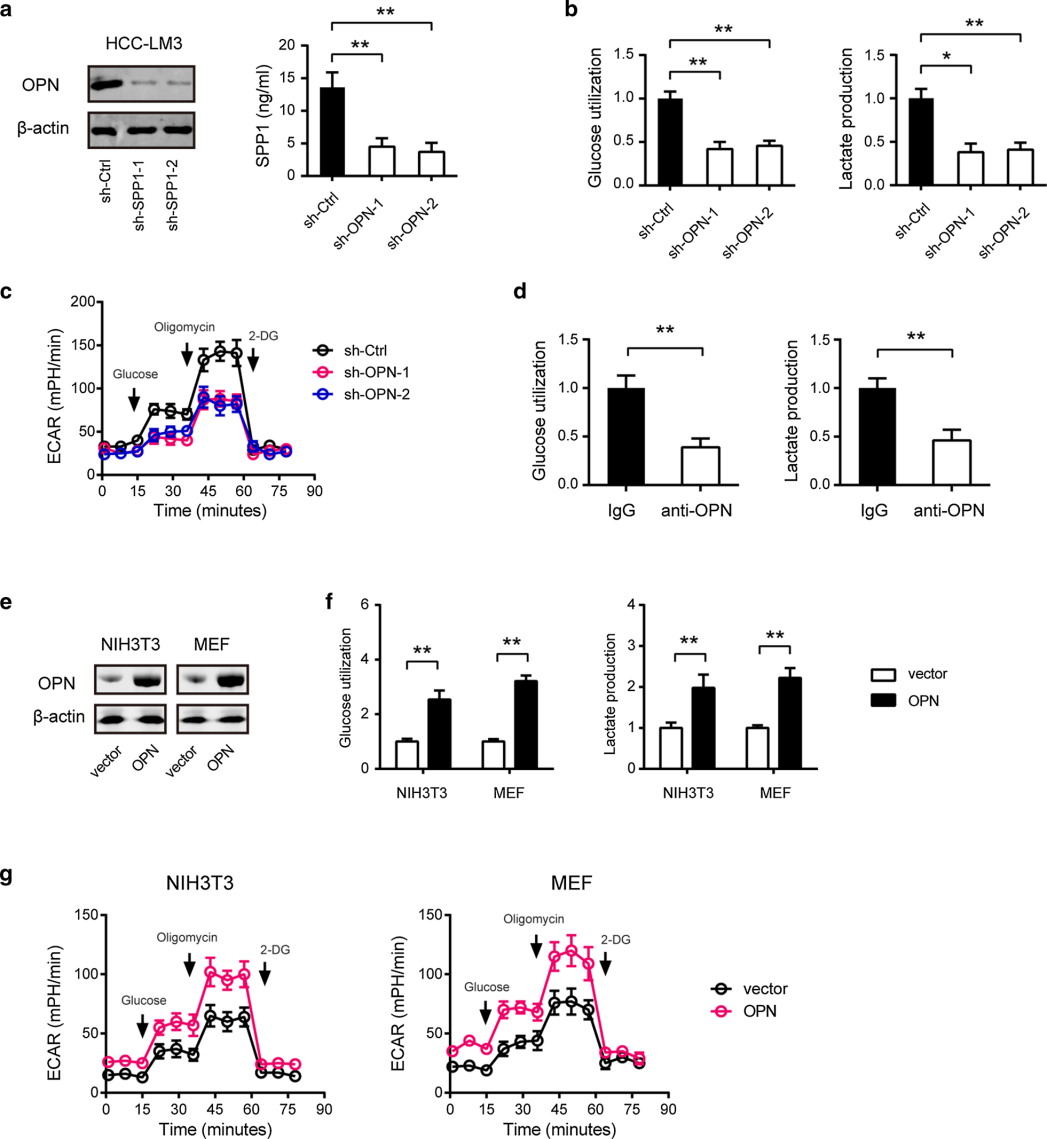
OPN promotes the Warburg effect in HCC cells. **a** The knockdown efficiency of OPN in HCC-LM3 cells was measured by Western blotting and ELISA. **b** Effects of OPN knockdown on the glucose uptake and lactate production in HCC-LM3 cells (*n* = 3). **c** The extracellular acidification rate (ECAR) in sh-OPN and sh-Ctrl HCC-LM3 cells was measured by Seahorse analyzer (*n* = 5). **d** Effects of OPN blockade on the glucose uptake and lactate production in HCC-LM3 cells (*n* = 3). **e** The overexpression efficiency of OPN in NIH3T3 cells and MEFs was measured by Western blotting. **f** Effects of OPN overexpression on the glucose uptake and lactate production in NIH3T3 cells and MEFs (*n* = 3). **g** Effects of OPN overexpression on ECAR in NIH3T3 cells and MEFs were measured by Seahorse analyzer (*n* = 5). *P < 0.05 and **P < 0.01

**Fig. 8:**
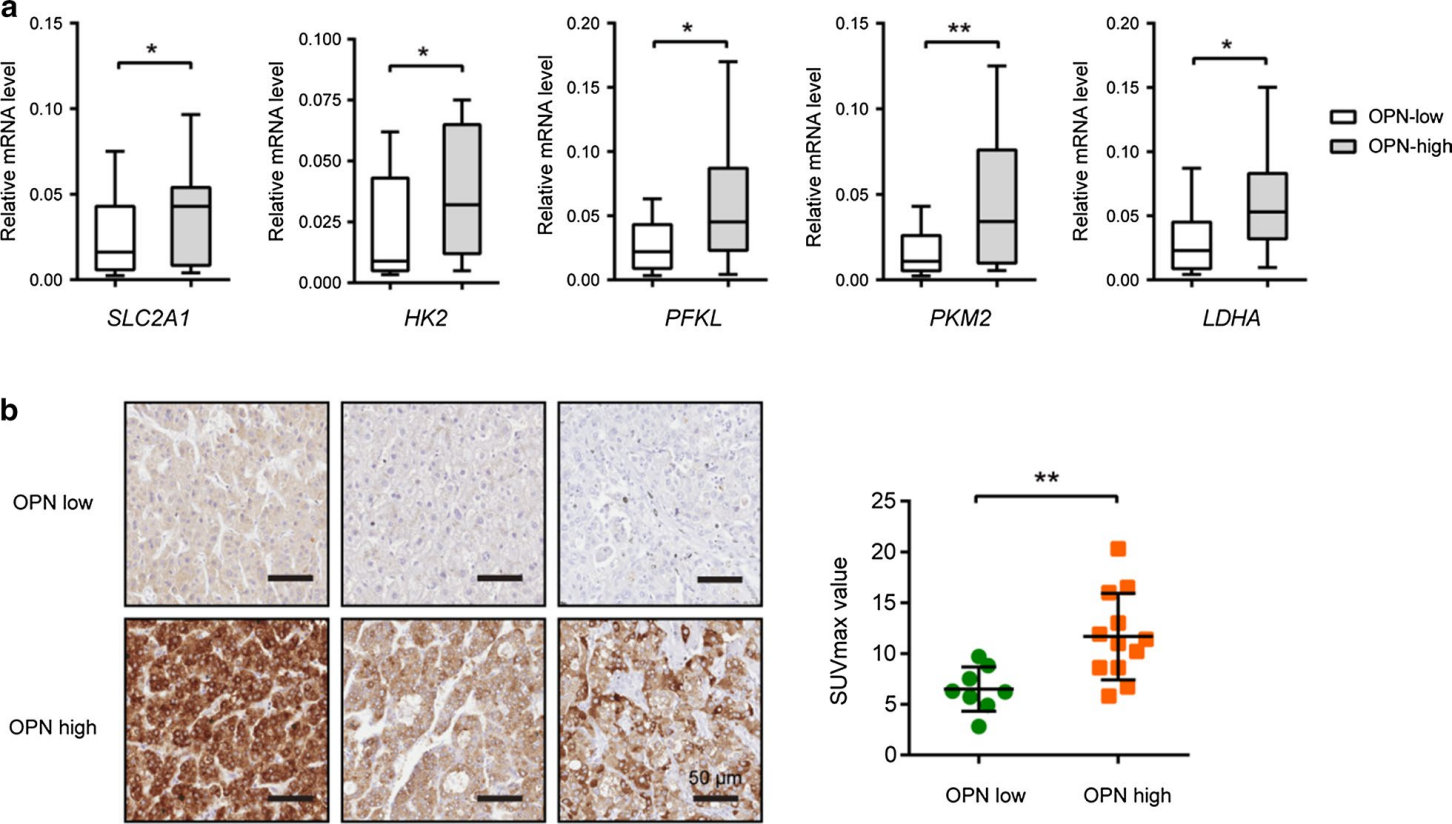
Expression pattern of OPN in clinical samples. **a** The expression of glycolytic genes in human HCC tissue samples with high OPN (*n* = 10) and low OPN (*n* = 20) expression was analyzed by real-time qPCR. **b** Representative photographs of OPN expression in HCC tumor tissues; scale bar: 50 μm. The correlation between OPN expression and the SUVmax value was analyzed. *P < 0.05 and **P < 0.01
