# Supplementary material for: Integrated analysis reveals critical glycolytic regulators in hepatocellular carcinoma
Source: Cell Commun Signal. 2020 Jun 23;18:97. doi: 10.1186/s12964-020-00539-4 (PMC7310503; doi:10.1186/s12964-020-00539-4)
Supplement: Supplementary file 2 — Additional file 1 Supplementary Fig. 1 The prognostic value of glycolysis-related genes in HCC. Supplementary Fig. 2 The correlation between glycolysis-related genes and the glycolysis signature. Supplementary Fig. 3 Expression pattern and roles of OPN in HCC cells. Supplementary Fig. 4 Expression pattern and prognostic value of OPN in HCC tissues. Supplementary Table 1: Primers used in this study. Supplementary Table 2: Group information. [file 12964_2020_539_MOESM2_ESM.docx]

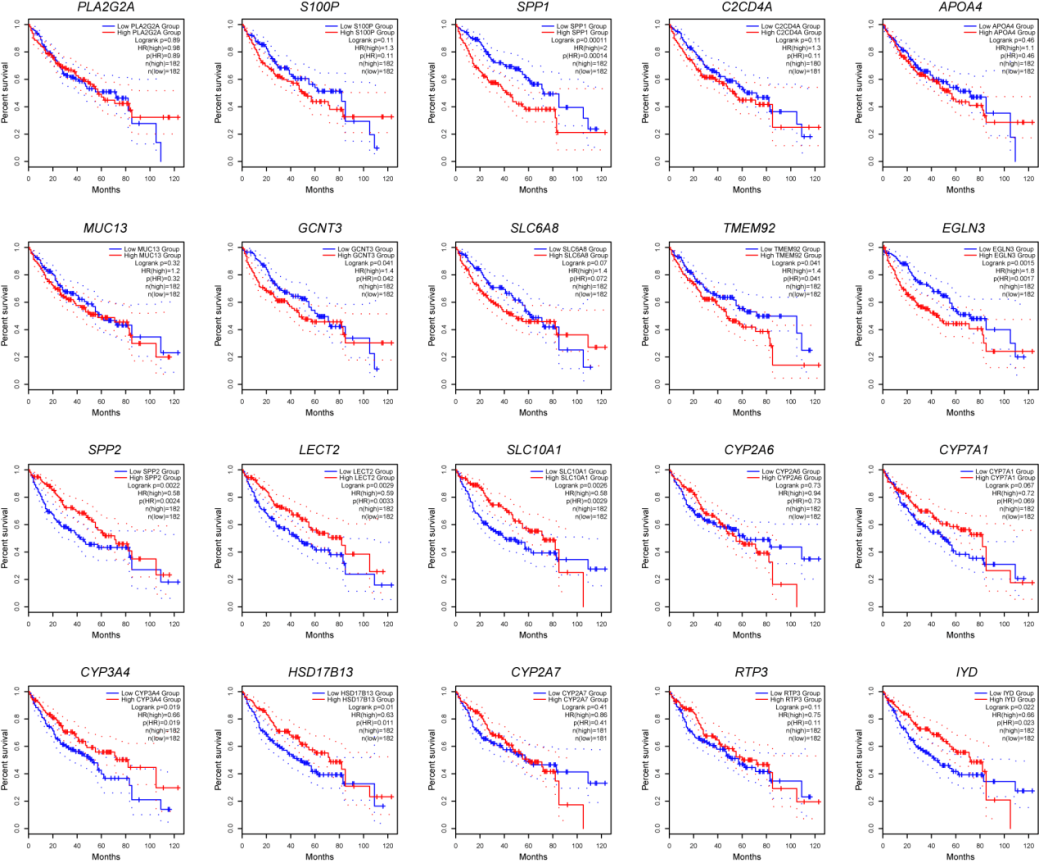


**Supplementary Fig. 1** The prognostic value of glycolysis-related genes in HCC.


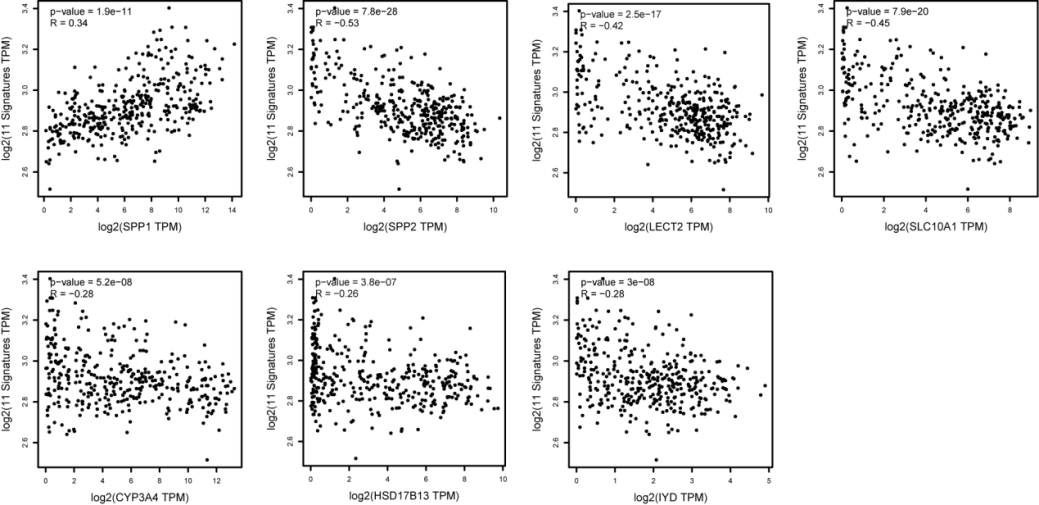


**Supplementary Fig. 2** The correlation between glycolysis-related genes and the glycolysis signature.


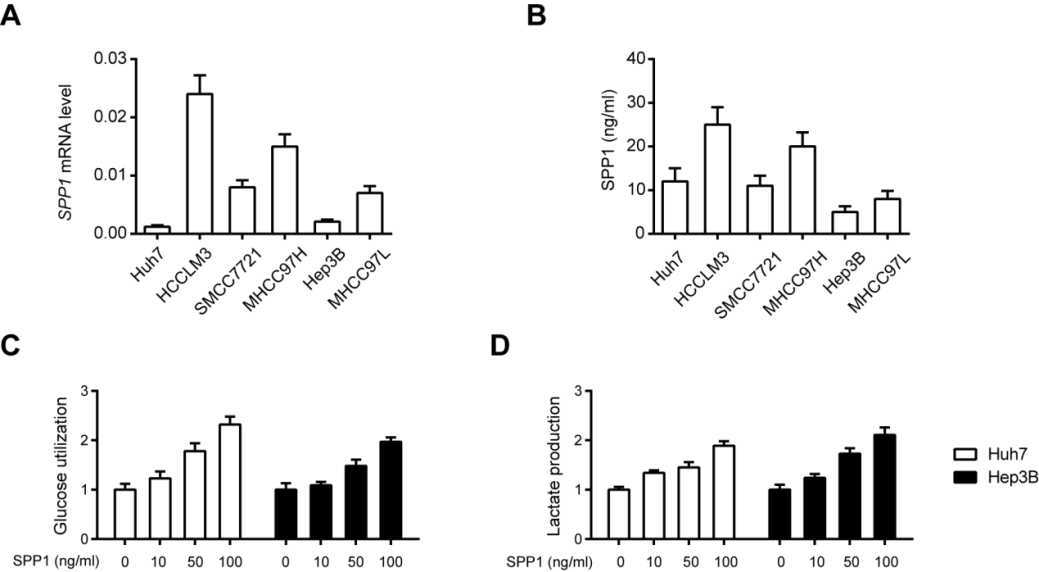


**Supplementary Fig. 3** Expression pattern and roles of OPN in HCC cells.

A. Real-time qPCR analysis of the mRNA level of OPN in HCC cell lines.

B. ELISA analysis of OPN in the culture supernatants of HCC cells.

C. The effect of OPN treatment on the glucose utilization of Huh7 and Hep3B cells.

D. The effect of OPN treatment on the lactate production of Huh7 and Hep3B cells.


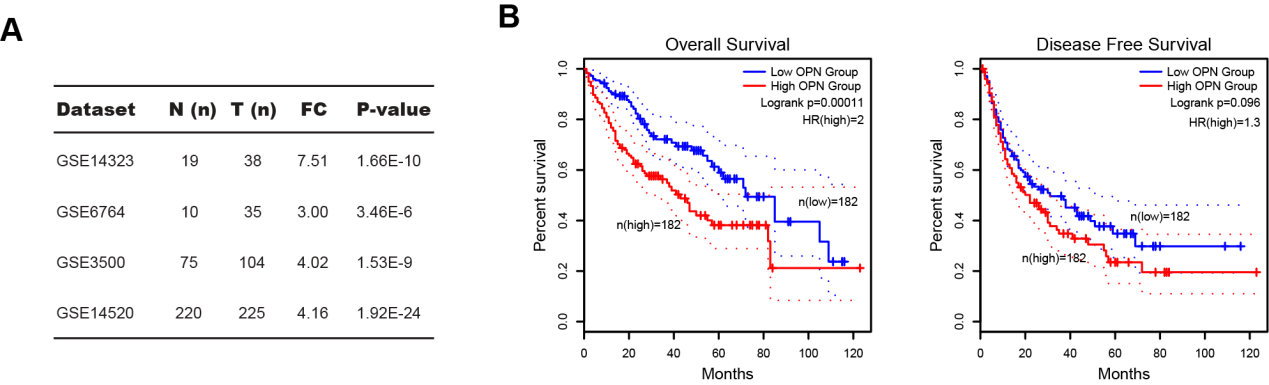


**Supplementary Fig. 4** Expression pattern and prognostic value of OPN in HCC tissues.

A. Oncomine database showed the overexpression profiles of OPN in HCC tissues compared to normal liver tissues.

B. The prognostic value of OPN in HCC was analyzed by the TCGA cohort.

**Supplementary table 1: Primers used in this study**

| Gene | Forward primer (5’-3’) | Reverse primer (5’-3’) |
| --- | --- | --- |
| *SPP2* | CCGTATCTGTTTCGGGCATTC | GGACACATAGTAGTCCCTCTGGA |
| *LECT2* | TGGGCCAGGAGAAACCTTATC | CAAGGGCAATAGAGTTCCAAGTT |
| *SLC10A1* | GGCCGTCACAGTTCTCTCTG | GGTGGCAATCAAGAGTGGTGT |
| *CYP3A4* | AAGTCGCCTCGAAGATACACA | CTGCTGGACATCAGGGTGAG |
| *HSD17B13* | TGGGTGATGTAACAATCGTGG | AGGTAAGGAATCCCTTCGTGG |
| *IYD* | CCAGACGTGAAGCACAAGATT | GGATGCCACAAGCGATGGAA |
| *SLC2A1* | ATTGGCTCCGGTATCGTCAAC | GCTCAGATAGGACATCCAGGGTA |
| *HK2* | GAGCCACCACTCACCCTACT | CCAGGCATTCGGCAATGTG |
| *PFKL* | GCTGGGCGGCACTATCATT | TCAGGTGCGAGTAGGTCCG |
| *PKM2* | ATAACGCCTACATGGAAAAGTGT | TAAGCCCATCATCCACGTAGA |
| *LDHA* | ATGGCAACTCTAAAGGATCAGC | CCAACCCCAACAACTGTAATCT |
| *ACTB* | CATGTACGTTGCTATCCAGGC | CTCCTTAATGTCACGCACGAT |

**Supplementary table 2: Group information.**

| Sample ID | Glycolysis score | Group |
| --- | --- | --- |
| TCGA-DD-A4NQ-01 | 0.927880739 | High |
| TCGA-GJ-A6C0-01 | 0.843285433 | High |
| TCGA-ED-A7PZ-01 | 0.817944455 | High |
| TCGA-G3-AAV7-01 | 0.797143145 | High |
| TCGA-DD-A39Y-01 | 0.795922314 | High |
| TCGA-RC-A6M5-01 | 0.76820108 | High |
| TCGA-ED-A459-01 | 0.760088877 | High |
| TCGA-CC-5263-01 | 0.750690773 | High |
| TCGA-DD-AACP-01 | 0.730275117 | High |
| TCGA-G3-A7M7-01 | 0.727283842 | High |
| TCGA-BC-4073-01 | 0.724347551 | High |
| TCGA-RC-A6M3-01 | 0.724091533 | High |
| TCGA-DD-A1EK-01 | 0.686929869 | High |
| TCGA-ES-A2HT-01 | 0.6853006 | High |
| TCGA-BC-A10U-01 | 0.685143194 | High |
| TCGA-CC-A3M9-01 | 0.675272354 | High |
| TCGA-2Y-A9H0-01 | 0.668977596 | High |
| TCGA-DD-A4NA-01 | 0.658027537 | High |
| TCGA-BC-A10Q-01 | 0.657885135 | High |
| TCGA-UB-A7MF-01 | 0.656190841 | High |
| TCGA-DD-AACL-01 | 0.648862056 | High |
| TCGA-DD-A3A4-01 | 0.648033724 | High |
| TCGA-2Y-A9GY-01 | 0.640542603 | High |
| TCGA-DD-AACX-01 | 0.636259594 | High |
| TCGA-G3-A25T-01 | 0.631510582 | High |
| TCGA-BC-A69H-01 | 0.629669415 | High |
| TCGA-CC-A7II-01 | 0.624825553 | High |
| TCGA-G3-A7M9-01 | 0.617136747 | High |
| TCGA-DD-A73B-01 | 0.61592808 | High |
| TCGA-YA-A8S7-01 | 0.613661354 | High |
| TCGA-DD-A1EJ-01 | 0.592591884 | High |
| TCGA-MR-A520-01 | 0.582790149 | High |
| TCGA-DD-AACB-01 | 0.568154698 | High |
| TCGA-FV-A3I0-01 | 0.567634158 | High |
| TCGA-DD-AACH-01 | 0.562074921 | High |
| TCGA-T1-A6J8-01 | 0.559723953 | High |
| TCGA-ZS-A9CD-01 | 0.555548493 | High |
| TCGA-G3-A25X-01 | 0.554540513 | High |
| TCGA-G3-A3CI-01 | 0.54724055 | High |
| TCGA-FV-A3I1-01 | 0.538096283 | High |
| TCGA-DD-A73G-01 | 0.530945879 | High |
| TCGA-CC-A7IJ-01 | 0.527671766 | High |
| TCGA-DD-A118-01 | 0.524652885 | High |
| TCGA-RC-A6M6-01 | 0.520526219 | High |
| TCGA-CC-A7IE-01 | 0.518309875 | High |
| TCGA-XR-A8TD-01 | 0.517988762 | High |
| TCGA-CC-A3MC-01 | 0.511759554 | High |
| TCGA-MI-A75C-01 | 0.503972858 | High |
| TCGA-ZP-A9D0-01 | 0.49982166 | High |
| TCGA-CC-5264-01 | 0.494324825 | High |
| TCGA-MI-A75I-01 | 0.492901671 | High |
| TCGA-DD-AACZ-01 | 0.492747085 | High |
| TCGA-BC-A110-01 | 0.487889199 | High |
| TCGA-5C-A9VG-01 | 0.486624133 | High |
| TCGA-FV-A4ZQ-01 | 0.481755514 | High |
| TCGA-DD-A3A1-01 | 0.477866555 | High |
| TCGA-FV-A4ZP-01 | 0.476918214 | High |
| TCGA-MR-A8JO-01 | 0.476304156 | High |
| TCGA-DD-A3A3-01 | 0.476246078 | High |
| TCGA-DD-AAEG-01 | 0.474218288 | High |
| TCGA-CC-A7IG-01 | 0.471180746 | High |
| TCGA-WX-AA47-01 | 0.469679415 | High |
| TCGA-DD-A4NR-01 | 0.464040124 | High |
| TCGA-DD-A1EL-01 | 0.46070504 | High |
| TCGA-ED-A7PX-01 | 0.460620158 | High |
| TCGA-DD-A113-01 | 0.455866806 | High |
| TCGA-DD-AAD0-01 | 0.4531936 | High |
| TCGA-KR-A7K7-01 | 0.449744591 | High |
| TCGA-CC-A8HU-01 | 0.438531221 | High |
| TCGA-DD-A1EC-01 | 0.438356543 | High |
| TCGA-DD-AAD5-01 | 0.437089234 | High |
| TCGA-BC-A8YO-01 | 0.41355789 | High |
| TCGA-DD-A1EE-01 | 0.41220278 | High |
| TCGA-ED-A82E-01 | 0.40528247 | High |
| TCGA-ED-A7XO-01 | 0.392274584 | High |
| TCGA-G3-A5SM-01 | 0.389533301 | High |
| TCGA-DD-AAD2-01 | 0.385763217 | High |
| TCGA-CC-A9FW-01 | 0.385215063 | High |
| TCGA-UB-A7MD-01 | 0.382228324 | High |
| TCGA-K7-AAU7-01 | 0.367814207 | High |
| TCGA-DD-A1EG-01 | 0.366574368 | High |
| TCGA-5R-AA1D-01 | 0.358762136 | High |
| TCGA-2Y-A9GU-01 | 0.356343706 | High |
| TCGA-DD-A116-01 | 0.352002897 | High |
| TCGA-BC-4072-01 | 0.351873128 | High |
| TCGA-K7-A6G5-01 | 0.351103679 | High |
| TCGA-CC-5259-01 | 0.341505246 | High |
| TCGA-CC-5262-01 | 0.339642609 | High |
| TCGA-DD-A39X-01 | 0.324793679 | High |
| TCGA-2Y-A9HA-01 | 0.321364698 | High |
| TCGA-DD-AAC8-01 | 0.319748451 | High |
| TCGA-CC-A9FV-01 | 0.317778219 | High |
| TCGA-DD-AAVP-01 | 0.317288783 | High |
| TCGA-DD-A4NB-01 | 0.305036762 | High |
| TCGA-DD-A114-01 | 0.303752222 | High |
| TCGA-CC-A8HT-01 | 0.297835694 | High |
| TCGA-CC-A5UE-01 | 0.296025993 | High |
| TCGA-DD-AAVX-01 | 0.294912514 | High |
| TCGA-DD-AAEI-01 | 0.289196896 | High |
| TCGA-BC-A10X-01 | 0.288622929 | High |
| TCGA-DD-A1EB-01 | 0.287873494 | High |
| TCGA-CC-5258-01 | 0.281920703 | High |
| TCGA-DD-AADM-01 | 0.275268189 | High |
| TCGA-BC-A5W4-01 | 0.273041573 | High |
| TCGA-FV-A2QQ-01 | 0.271193398 | High |
| TCGA-CC-A1HT-01 | 0.267904257 | High |
| TCGA-MI-A75G-01 | 0.267211 | High |
| TCGA-GJ-A3OU-01 | 0.259353056 | High |
| TCGA-2Y-A9H8-01 | 0.251835773 | High |
| TCGA-RC-A6M4-01 | 0.237823332 | High |
| TCGA-FV-A3R3-01 | 0.232889675 | High |
| TCGA-G3-A25S-01 | 0.22046765 | High |
| TCGA-BD-A3ER-01 | 0.219635902 | High |
| TCGA-DD-AAVU-01 | 0.218730108 | High |
| TCGA-DD-AAC9-01 | 0.218488202 | High |
| TCGA-DD-A4NL-01 | 0.216620009 | High |
| TCGA-HP-A5MZ-01 | 0.21105674 | High |
| TCGA-G3-AAV6-01 | 0.200377453 | High |
| TCGA-G3-A25Z-01 | 0.196400992 | High |
| TCGA-EP-A2KA-01 | 0.194928537 | High |
| TCGA-DD-AADN-01 | 0.194444965 | High |
| TCGA-5C-AAPD-01 | 0.193244028 | High |
| TCGA-G3-A5SJ-01 | 0.185141628 | High |
| TCGA-ED-A66X-01 | 0.179139904 | High |
| TCGA-G3-A25V-01 | 0.173245878 | High |
| TCGA-RC-A7SB-01 | 0.166950802 | High |
| TCGA-CC-A9FU-01 | 0.162895483 | High |
| TCGA-G3-AAV4-01 | 0.15432451 | High |
| TCGA-DD-A3A2-01 | 0.154244555 | High |
| TCGA-2Y-A9GW-01 | 0.151053087 | High |
| TCGA-G3-A7M8-01 | 0.150636328 | High |
| TCGA-G3-A5SI-01 | 0.139024624 | High |
| TCGA-DD-A119-01 | 0.136758374 | High |
| TCGA-DD-A3A7-01 | 0.134347629 | High |
| TCGA-NI-A4U2-01 | 0.133248682 | High |
| TCGA-DD-AADQ-01 | 0.13205994 | High |
| TCGA-2Y-A9H2-01 | 0.130687541 | High |
| TCGA-G3-A3CG-01 | 0.130015592 | High |
| TCGA-2Y-A9H9-01 | 0.126578792 | High |
| TCGA-ZS-A9CG-01 | 0.121532705 | High |
| TCGA-DD-AAVV-01 | 0.120942411 | High |
| TCGA-K7-A5RF-01 | 0.120630519 | High |
| TCGA-LG-A9QC-01 | 0.118931039 | High |
| TCGA-DD-AAW1-01 | 0.117293765 | High |
| TCGA-DD-A4NH-01 | 0.112235052 | High |
| TCGA-DD-A3A8-01 | 0.108665809 | High |
| TCGA-G3-A3CJ-01 | 0.108373348 | High |
| TCGA-G3-A5SK-01 | 0.108020137 | High |
| TCGA-G3-AAV5-01 | 0.104235825 | High |
| TCGA-RC-A7S9-01 | 0.100339869 | High |
| TCGA-MI-A75E-01 | 0.096446145 | High |
| TCGA-DD-A11A-01 | 0.092607479 | High |
| TCGA-DD-A11D-01 | 0.089382212 | High |
| TCGA-BW-A5NQ-01 | 0.087645041 | High |
| TCGA-DD-A73F-01 | 0.086523619 | High |
| TCGA-GJ-A9DB-01 | 0.08549081 | High |
| TCGA-DD-AAEK-01 | 0.085172128 | High |
| TCGA-BC-A112-01 | 0.077251571 | High |
| TCGA-O8-A75V-01 | 0.065825002 | High |
| TCGA-DD-A1ED-01 | 0.06335161 | High |
| TCGA-5R-AAAM-01 | 0.059982557 | High |
| TCGA-BC-A10W-01 | 0.059642345 | High |
| TCGA-ED-A627-01 | 0.05701197 | High |
| TCGA-RC-A7SK-01 | 0.039832482 | High |
| TCGA-BC-A69I-01 | 0.039442436 | High |
| TCGA-DD-A4NE-01 | 0.037583011 | High |
| TCGA-2Y-A9H3-01 | 0.03728854 | High |
| TCGA-DD-AADB-01 | 0.033350361 | High |
| TCGA-G3-A7M5-01 | 0.03234102 | High |
| TCGA-DD-A3A9-01 | 0.029774502 | High |
| TCGA-FV-A3R2-01 | 0.02577196 | High |
| TCGA-ED-A97K-01 | 0.022382293 | High |
| TCGA-DD-A73C-01 | 0.017343757 | High |
| TCGA-DD-AADO-01 | 0.015252135 | High |
| TCGA-DD-A4NO-01 | 0.007207253 | High |
| TCGA-DD-A3A5-01 | 0.007087325 | High |
| TCGA-DD-AACG-01 | -0.001342992 | High |
| TCGA-DD-AAW0-01 | -0.007808509 | High |
| TCGA-QA-A7B7-01 | -0.015179158 | High |
| TCGA-EP-A3JL-01 | -0.016278949 | High |
| TCGA-DD-AACD-01 | -0.016930178 | High |
| TCGA-ZS-A9CE-01 | -0.02126037 | High |
| TCGA-2Y-A9HB-01 | -0.027738177 | High |
| TCGA-DD-AAVR-01 | -0.028469778 | High |
| TCGA-ZP-A9CZ-01 | -0.035517885 | High |
| TCGA-DD-A3A6-01 | -0.048431701 | Low |
| TCGA-DD-AACF-01 | -0.056098088 | Low |
| TCGA-ZP-A9CV-01 | -0.061593962 | Low |
| TCGA-DD-AAVY-01 | -0.061961687 | Low |
| TCGA-CC-5260-01 | -0.06474248 | Low |
| TCGA-BC-A10R-01 | -0.076916089 | Low |
| TCGA-G3-A3CK-01 | -0.083339415 | Low |
| TCGA-FV-A496-01 | -0.087154137 | Low |
| TCGA-FV-A495-01 | -0.091295346 | Low |
| TCGA-DD-AA3A-01 | -0.091583107 | Low |
| TCGA-CC-A7IL-01 | -0.094837952 | Low |
| TCGA-BW-A5NP-01 | -0.106978397 | Low |
| TCGA-CC-A5UC-01 | -0.111395218 | Low |
| TCGA-ZP-A9D1-01 | -0.113287262 | Low |
| TCGA-DD-AACT-01 | -0.11469123 | Low |
| TCGA-BC-A10S-01 | -0.115129584 | Low |
| TCGA-DD-AACK-01 | -0.116018995 | Low |
| TCGA-EP-A3RK-01 | -0.121305247 | Low |
| TCGA-ES-A2HS-01 | -0.128516124 | Low |
| TCGA-DD-AADK-01 | -0.130428099 | Low |
| TCGA-2Y-A9H1-01 | -0.130482376 | Low |
| TCGA-DD-AADF-01 | -0.130701306 | Low |
| TCGA-DD-AADS-01 | -0.133255491 | Low |
| TCGA-DD-AAE3-01 | -0.134423119 | Low |
| TCGA-DD-AADJ-01 | -0.135877939 | Low |
| TCGA-BC-A217-01 | -0.136977304 | Low |
| TCGA-BC-A10Z-01 | -0.145654731 | Low |
| TCGA-G3-AAV3-01 | -0.148398 | Low |
| TCGA-LG-A9QD-01 | -0.15194692 | Low |
| TCGA-DD-A4NP-01 | -0.152691033 | Low |
| TCGA-LG-A6GG-01 | -0.154351628 | Low |
| TCGA-KR-A7K8-01 | -0.157687356 | Low |
| TCGA-DD-AACC-01 | -0.162158633 | Low |
| TCGA-DD-A1EA-01 | -0.167214737 | Low |
| TCGA-BD-A3EP-01 | -0.177583832 | Low |
| TCGA-2Y-A9GS-01 | -0.178549182 | Low |
| TCGA-DD-A4NF-01 | -0.181796754 | Low |
| TCGA-RC-A7SF-01 | -0.182412175 | Low |
| TCGA-ED-A8O6-01 | -0.201695318 | Low |
| TCGA-ZP-A9CY-01 | -0.204407032 | Low |
| TCGA-PD-A5DF-01 | -0.210574547 | Low |
| TCGA-DD-AAD3-01 | -0.216050237 | Low |
| TCGA-RG-A7D4-01 | -0.221316278 | Low |
| TCGA-ED-A7XP-01 | -0.222340297 | Low |
| TCGA-CC-A3MA-01 | -0.223301863 | Low |
| TCGA-ED-A4XI-01 | -0.231933317 | Low |
| TCGA-KR-A7K0-01 | -0.232731566 | Low |
| TCGA-2V-A95S-01 | -0.241552222 | Low |
| TCGA-FV-A2QR-01 | -0.245168325 | Low |
| TCGA-5R-AA1C-01 | -0.257083645 | Low |
| TCGA-DD-AACI-01 | -0.258084964 | Low |
| TCGA-DD-AACU-01 | -0.258343171 | Low |
| TCGA-DD-A4NK-01 | -0.270272677 | Low |
| TCGA-DD-AACS-01 | -0.273572486 | Low |
| TCGA-DD-AAE6-01 | -0.27892595 | Low |
| TCGA-DD-AAE4-01 | -0.28081412 | Low |
| TCGA-DD-A4NV-01 | -0.283168258 | Low |
| TCGA-CC-A3MB-01 | -0.28329642 | Low |
| TCGA-ED-A5KG-01 | -0.28586355 | Low |
| TCGA-DD-A1EI-01 | -0.287772925 | Low |
| TCGA-EP-A12J-01 | -0.289223582 | Low |
| TCGA-XR-A8TG-01 | -0.291467702 | Low |
| TCGA-G3-AAV1-01 | -0.294338624 | Low |
| TCGA-BC-A3KF-01 | -0.296298529 | Low |
| TCGA-DD-A4NN-01 | -0.302016154 | Low |
| TCGA-3K-AAZ8-01 | -0.303865513 | Low |
| TCGA-DD-AAVQ-01 | -0.304345765 | Low |
| TCGA-DD-AACJ-01 | -0.306353214 | Low |
| TCGA-G3-A3CH-01 | -0.30646588 | Low |
| TCGA-BC-A3KG-01 | -0.311102462 | Low |
| TCGA-BC-A10T-01 | -0.318102536 | Low |
| TCGA-G3-AAV2-01 | -0.324163496 | Low |
| TCGA-2Y-A9GV-01 | -0.328896249 | Low |
| TCGA-4R-AA8I-01 | -0.330243683 | Low |
| TCGA-K7-A5RG-01 | -0.331619096 | Low |
| TCGA-DD-A39V-01 | -0.33505049 | Low |
| TCGA-DD-AACE-01 | -0.335267008 | Low |
| TCGA-CC-A7IK-01 | -0.337252055 | Low |
| TCGA-DD-AACY-01 | -0.338837368 | Low |
| TCGA-XR-A8TE-01 | -0.343715537 | Low |
| TCGA-DD-AAEE-01 | -0.346261632 | Low |
| TCGA-G3-AAUZ-01 | -0.34749964 | Low |
| TCGA-WQ-A9G7-01 | -0.358666548 | Low |
| TCGA-CC-A5UD-01 | -0.358865126 | Low |
| TCGA-WX-AA46-01 | -0.360779554 | Low |
| TCGA-DD-AAE2-01 | -0.363012405 | Low |
| TCGA-G3-AAV0-01 | -0.372693279 | Low |
| TCGA-DD-A73A-01 | -0.373160456 | Low |
| TCGA-DD-A4NS-01 | -0.374112697 | Low |
| TCGA-DD-AACQ-01 | -0.376352414 | Low |
| TCGA-DD-A115-01 | -0.380572016 | Low |
| TCGA-2Y-A9H4-01 | -0.381967195 | Low |
| TCGA-EP-A26S-01 | -0.388032928 | Low |
| TCGA-MI-A75H-01 | -0.394126394 | Low |
| TCGA-DD-AADG-01 | -0.3972176 | Low |
| TCGA-DD-AAE9-01 | -0.397498954 | Low |
| TCGA-DD-A11C-01 | -0.397986175 | Low |
| TCGA-2Y-A9GT-01 | -0.401756666 | Low |
| TCGA-DD-AADV-01 | -0.405706722 | Low |
| TCGA-ZP-A9D2-01 | -0.408814179 | Low |
| TCGA-DD-A4NG-01 | -0.411870436 | Low |
| TCGA-UB-A7MA-01 | -0.418538364 | Low |
| TCGA-UB-A7MC-01 | -0.421367443 | Low |
| TCGA-DD-AAE0-01 | -0.421677014 | Low |
| TCGA-2Y-A9GX-01 | -0.433227917 | Low |
| TCGA-DD-AADC-01 | -0.434763301 | Low |
| TCGA-G3-A25Y-01 | -0.441851629 | Low |
| TCGA-2Y-A9GZ-01 | -0.447643843 | Low |
| TCGA-DD-A4NJ-01 | -0.450078912 | Low |
| TCGA-UB-AA0V-01 | -0.45065782 | Low |
| TCGA-G3-A5SL-01 | -0.451867776 | Low |
| TCGA-DD-A39Z-01 | -0.452566554 | Low |
| TCGA-DD-AADP-01 | -0.453125665 | Low |
| TCGA-DD-A11B-01 | -0.45392596 | Low |
| TCGA-WQ-AB4B-01 | -0.45897983 | Low |
| TCGA-ZS-A9CF-01 | -0.462951247 | Low |
| TCGA-RC-A7SH-01 | -0.464316938 | Low |
| TCGA-NI-A8LF-01 | -0.465068251 | Low |
| TCGA-BW-A5NO-01 | -0.468068397 | Low |
| TCGA-DD-AAVZ-01 | -0.471002807 | Low |
| TCGA-XR-A8TC-01 | -0.471678207 | Low |
| TCGA-DD-A4ND-01 | -0.473083784 | Low |
| TCGA-CC-A7IH-01 | -0.474087631 | Low |
| TCGA-G3-A6UC-01 | -0.479396295 | Low |
| TCGA-CC-A9FS-01 | -0.480231731 | Low |
| TCGA-UB-A7MB-01 | -0.480362142 | Low |
| TCGA-DD-AADD-01 | -0.492395394 | Low |
| TCGA-UB-AA0U-01 | -0.510164217 | Low |
| TCGA-CC-A123-01 | -0.51138516 | Low |
| TCGA-DD-AADI-01 | -0.513526091 | Low |
| TCGA-DD-AACA-01 | -0.514850117 | Low |
| TCGA-DD-AADR-01 | -0.515222904 | Low |
| TCGA-DD-AAE7-01 | -0.517819983 | Low |
| TCGA-UB-A7ME-01 | -0.521731295 | Low |
| TCGA-DD-AAVW-01 | -0.522959957 | Low |
| TCGA-DD-AAED-01 | -0.526162401 | Low |
| TCGA-CC-A7IF-01 | -0.528789316 | Low |
| TCGA-BC-A216-01 | -0.530780403 | Low |
| TCGA-DD-A1EF-01 | -0.53583726 | Low |
| TCGA-DD-AAEA-01 | -0.536607456 | Low |
| TCGA-XR-A8TF-01 | -0.536788509 | Low |
| TCGA-DD-AACW-01 | -0.538354798 | Low |
| TCGA-G3-A25U-01 | -0.538393158 | Low |
| TCGA-DD-AACV-01 | -0.538453933 | Low |
| TCGA-DD-A73E-01 | -0.539787617 | Low |
| TCGA-BC-A10Y-01 | -0.545451662 | Low |
| TCGA-DD-A39W-01 | -0.550921385 | Low |
| TCGA-DD-AADL-01 | -0.557484693 | Low |
| TCGA-DD-AAW2-01 | -0.560525024 | Low |
| TCGA-DD-AADU-01 | -0.561677727 | Low |
| TCGA-DD-AAEH-01 | -0.565266987 | Low |
| TCGA-DD-AAD1-01 | -0.569516268 | Low |
| TCGA-DD-AADY-01 | -0.571379803 | Low |
| TCGA-DD-AADW-01 | -0.582252274 | Low |
| TCGA-DD-A4NI-01 | -0.583096521 | Low |
| TCGA-DD-AAD8-01 | -0.587341982 | Low |
| TCGA-WX-AA44-01 | -0.592899333 | Low |
| TCGA-CC-5261-01 | -0.59391654 | Low |
| TCGA-WJ-A86L-01 | -0.594878413 | Low |
| TCGA-DD-AACO-01 | -0.604829745 | Low |
| TCGA-DD-A73D-01 | -0.608333941 | Low |
| TCGA-5C-A9VH-01 | -0.608543427 | Low |
| TCGA-CC-A8HS-01 | -0.610746028 | Low |
| TCGA-ED-A66Y-01 | -0.611150734 | Low |
| TCGA-G3-A7M6-01 | -0.61622624 | Low |
| TCGA-DD-AAE1-01 | -0.628959218 | Low |
| TCGA-ZP-A9D4-01 | -0.635414327 | Low |
| TCGA-DD-A1EH-01 | -0.636199822 | Low |
| TCGA-DD-AACN-01 | -0.638987533 | Low |
| TCGA-ED-A8O5-01 | -0.642696839 | Low |
| TCGA-EP-A2KC-01 | -0.643362767 | Low |
| TCGA-2Y-A9H6-01 | -0.64835924 | Low |
| TCGA-DD-AAW3-01 | -0.650505802 | Low |
| TCGA-EP-A2KB-01 | -0.650533847 | Low |
| TCGA-2Y-A9H5-01 | -0.653711493 | Low |
| TCGA-2Y-A9H7-01 | -0.658979785 | Low |
| TCGA-DD-AAD6-01 | -0.661691817 | Low |
| TCGA-HP-A5N0-01 | -0.664390983 | Low |
| TCGA-ED-A7PY-01 | -0.671518614 | Low |
| TCGA-DD-AADA-01 | -0.712123586 | Low |
| TCGA-BD-A2L6-01 | -0.726193238 | Low |
| TCGA-FV-A23B-01 | -0.741556361 | Low |
| TCGA-CC-A8HV-01 | -0.750455138 | Low |
| TCGA-KR-A7K2-01 | -0.765617401 | Low |
| TCGA-DD-AAEB-01 | -0.775781592 | Low |
| TCGA-DD-AAVS-01 | -0.805751741 | Low |
